# Bi-allelic *RNU6ATAC* variants cause a minor spliceopathy characterized by transcriptome-wide minor intron retention and multisystem manifestations

**DOI:** 10.1016/j.xhgg.2026.100588

**Published:** 2026-03-09

**Authors:** Rodrigo Mendez, Taylor M. Arriaga, Jialan Ma, Devon E. Bonner, Sara Emami, Rebecca J. Levy, Afaf Alsagheir, Bader Alhaddad, Khadijah Bakur, Rachel A. Ungar, Dena R. Matalon, Alexander M. Miller, Jonathan Nguyen, Kevin S. Smith, Stuart A. Scott, Linda Liao, Zena Ng, Shruti Marwaha, Alistair Ward, Undiagnosed Diseases Network, Genomics Research to Elucidate the Genetics of Rare Diseases Consortium, Danica Novacic, Fowzan S. Alkuraya, Jonathan A. Bernstein, Vijay S. Ganesh, Anne O’Donnell-Luria, Stephen B. Montgomery, Matthew T. Wheeler

**Affiliations:** 1 Division of Cardiovascular Medicine, Department of Medicine, Stanford University, Stanford, CA, USA; 2 Department of Genetics, Stanford University, Stanford, CA, USA; 3 Program in Medical and Population Genetics, Broad Institute of MIT and Harvard, Cambridge, MA, USA; 4 Division of Medical Genetics, Department of Pediatrics, Stanford University School of Medicine, Stanford, CA, USA; 5 Division of Child Neurology, Department of Neurology and Neurological Sciences, Stanford University, Stanford, CA, USA; 6 Department of Pediatrics, King Faisal Specialist Hospital and Research Centre, Riyadh, Saudi Arabia; 7 College of Medicine, Alfaisal University, Riyadh, Saudi Arabia; 8 Lifera Omics, Riyadh, Saudi Arabia; 9 Stanford Center for Biomedical Ethics, Stanford University, Stanford, CA, USA; 10 Department of Pathology, Stanford University, Stanford, CA, USA; 11 Clinical Genomics Laboratory, Stanford Medicine, Stanford, CA, USA; 12 Department of Human Genetics, University of Utah, Salt Lake City, UT, USA; 13 Frameshift Labs, Cambridge, MA, USA; 14 Undiagnosed Diseases Program, National Human Genome Research Institute, National Institutes of Health, Bethesda, MD, USA; 15 Department of Translational Genomics, Genomic Medicine Center of Excellence, King Faisal Specialist Hospital and Research Center, Riyadh, Saudi Arabia; 16 Department of Neurology, Brigham and Women’s Hospital, Boston, MA, USA; 17 Division of Genetics and Genomics, Boston Children’s Hospital, Boston, MA, USA

**Keywords:** bioinformatics, transcriptome-wide, minor spliceosome, RNU6ATAC, RNU4ATAC, minor intron retention, minor intron-containing genes

## Abstract

We report three individuals with bi-allelic variants in RNU6ATAC, which encodes the U6atac minor spliceosomal small nuclear RNA (snRNA), causing a multisystem minor spliceopathy. Through RNA sequencing analysis, we identified a distinctive excess of minor intron retention (MIR) in two unrelated individuals, which guided the identification of bi-allelic *RNU6ATAC* variants. The discovery cohort presented with variable multisystem manifestations. One individual presented with refractory epilepsy, microcephaly, developmental delay, ataxia, bilateral toe syndactyly, hypereosinophilia, and short stature, whereas the other exhibited failure to thrive, short stature, primary hypothyroidism, combined variable immunodeficiency, eosinophilic colitis, ichthyosis vulgaris, scoliosis, and chronic inflammatory demyelinating polyneuropathy without neurodevelopmental involvement. Despite organ-specific variation, both individuals displayed impaired growth and eosinophil-driven inflammation. Recently, we identified a third affected individual from an independent cohort whose phenotype bridges these features, combining microcephaly, growth failure with severe immunodeficiency, and skeletal abnormalities. The distinctive excess of MIR outliers in the discovery cohort supports minor spliceosome dysfunction, mirroring the molecular signature of RNU4ATAC-opathy. These findings nominate *RNU6ATAC* as a disease-associated gene, defining an expanded clinical spectrum of minor spliceopathies. Our study supports the power of integrating genomic and transcriptomic approaches for diagnosing splicing disorders and highlights the critical role of spliceosomal snRNAs in human disease.

## Teaser

By combining genomic and transcriptomic analyses, we provide evidence that bi-allelic variants in *RNU6ATAC*, a minor spliceosomal small nuclear RNA (snRNA), cause a minor spliceopathy characterized by minor intron retention, growth failure, and multisystem disease, thereby expanding the spectrum of minor spliceopathies and underscoring snRNA dysfunction as a cause of human disease.

## Introduction

Humans have two spliceosome machineries, characterized by unique consensus sequences and ribonucleoprotein (RNP) complexes.^1^ The major spliceosome removes over 99.5% of introns, and the minor spliceosome processes the remaining <0.5% (∼770 introns in 715 essential genes).^1^ The minor spliceosome comprises five small nuclear RNAs (snRNAs): U11, U12, U4atac, U6atac, and U5, the latter of which is shared with the major spliceosome. U11 and U12 form a stable U11/U12 di-snRNP that recognizes the minor 5′ splice site and branchpoint sequence early in spliceosome assembly.^2^ U4atac and U6atac create a bimolecule through extensive base pairing in the conserved stem I and II regions of U4atac.^3^^,^^4^ Disruption of U4atac destabilizes this complex, preventing proper trimolecule assembly with U5, and thereby compromising minor intron recognition and processing.^5^ Once activated, U6atac dissociates from U4atac and establishes new base-pairing interactions with U12 and the branchpoint of the intron, forming the catalytic core of the spliceosome.^6^ Overall, the integrity of each snRNA is essential for minor intron splicing, and disruption in any of these components could result in minor intron retention (MIR).^7^

Pathogenic variants in minor spliceosomal snRNAs have been identified in *RNU4ATAC*^8^ (RNA, U4atac small nuclear [MIM: 601428]) and *RNU12*^9^ (RNA, U12 small nuclear [MIM: 620204]). Bi-allelic variants in *RNU4ATAC* were reported in microcephalic osteodysplastic primordial dwarfism type 1 (MOPD1 [MIM: 210710]), Roifman (RFMN [MIM: 616651]), and Lowry-Wood (LWS [MIM: 226960]) syndromes,^10^ which are now considered RNU4atac-opathies. These disorders encompass a clinical spectrum, including severe short stature or spondyloepiphyseal dysplasia, microcephaly, neurodevelopmental delay, retinal dystrophy, and immunodeficiency.^11^ Bi-allelic variants in *RNU12* cause autosomal recessive spinocerebellar ataxia-33^9^ (SCAR33 [MIM: 620208]), and CDAGS syndrome ([MIM: 603116]) (an acronym for craniosynostosis, delayed fontanel closure, anal anomalies, genitourinary, and skin abnormalities).^12^ These disorders illustrate how defects in the minor spliceosome lead to a broad array of clinical phenotypes, from multisystem skeletal dysplasia with brain and immune involvement to more organ-restricted effects, as seen in SCAR33.

In this study, we identified bi-allelic *RNU6ATAC* (RNA, U6atac small nuclear [MIM: 601429]) variants in three unrelated individuals, guided by MIR outliers in the discovery cohort. We aim to demonstrate that *RNU6ATAC* variants cause transcriptome-wide minor splicing disruption and correlate this dysfunction with the observed phenotypes.

## Material and methods

### Ethics declaration

Undiagnosed Diseases Network (UDN) participants were enrolled under the Stanford University institutional review board (IRB) (protocols 47026 and 60837) and the National Human Genome Research Institute IRB (protocol 15-HG-0130). All individuals provided written informed consent. The replication cohort was enrolled under King Faisal Specialist Hospital and Research Center (KFSHRC) IRB approval (2230016), with consent for publication of identifiable information obtained under protocol Research Advisory Council (RAC) number 2080006.

### Transcriptome analysis

RNA sequencing (RNA-seq) methods for 385 whole-blood^5^^,^^13^ and 139 fibroblast samples are detailed in supplemental material 1. We adapted the outlier detection framework described in Arriaga et al.^5^ to specifically target MIR outliers. This refinement was motivated by the distinct architecture of minor intron-containing genes (MIGs), which typically contain multiple introns,^1^ only a small fraction of which are minor (U12-type) introns.^1^ Given that minor spliceopathies are characterized by the retention of minor introns,^14^ we refined our analysis to filter out canonical intron retention events within MIGs.

We applied FRASER^15^ separately to the whole-blood and fibroblast cohorts. Outliers were filtered to retain only significant *θ* outliers (representing partial or full intron retention) that met the significance thresholds established in Arriaga et al.^5^ We then quantified significant *θ* outliers in introns classified as “minor” in the Minor Intron Database,^1^ thereby calculating the number of MIR outliers per individual. Individuals were defined as having an excess of MIR if their count of MIR outliers was greater than 2 standard deviations from the mean of their respective tissue type.

The code used to filter, create, and analyze FRASER^15^ outliers is available in the FRASER_snakemake and run_results_phenotype_paper (https://github.com/maurermaggie/Transcriptome_Wide_Splicing_Analysis).

### Genome sequencing and reanalysis

Whole-genome sequencing (WGS) was performed for individuals A1 and B1 and their respective family members by Baylor Genetics through the UDN. Individual C1 and his parents underwent clinical WGS at Centogene, as requested by KFSHRC. Additional details can be found in supplemental material 1. WGS was considered essential for this study because *RNU6ATAC* is not targeted by major commercial whole-exome sequencing (WES) kits (according to the University of California, Santa Cruz [UCSC]^16^ “Exome Capture Probesets” track; GRCh38, chr9:134,164,439–134,164,564), and this was confirmed by the absence of sequencing coverage across the aggregated exome dataset in gnomAD.^17^^,^^18^ For the discovery cohort, genomic reanalysis was conducted using the Mosaic genomic platform (https://frameshift.io/mosaic) on the latest UDN dataset, which included WGS data from 5,323 individuals. Variant prioritization focused on rare variants (allele frequency [AF] <1% in gnomAD version 4.1.0^17^^,^^18^) located in minor spliceosome snRNAs.

## Results

### Transcriptome analysis

A recent study by Arriaga et al.^5^ employed FRASER^15^ to examine transcriptome-wide outlier patterns in 385 whole-blood samples from the UDN and Genomics Research to Elucidate the Genetics of Rare diseases (GREGoR) consortium. This approach identified five individuals with an excess of intron retention events in MIGs (mean: 286.6, median: 270, interquartile range [IQR]: 252–313) compared with the rest of the cohort (mean: 1.7, median: 0, IQR: 0–2), including six close relatives of the five aforementioned individuals. Genomic analysis of the five individuals with an excess of intron retention outliers in MIGs revealed that four had RNU4atac-opathies. The fifth individual (A1), who remained undiagnosed, had 252 intron retention events in MIGs.

**Figure 1:**
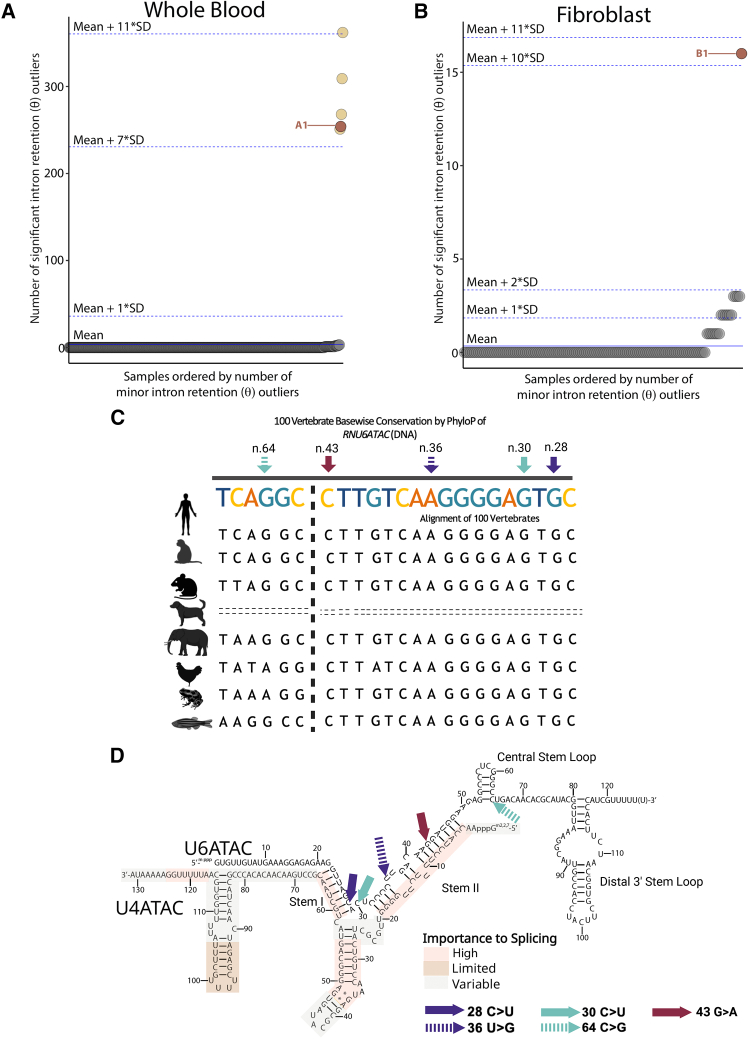
Rare, conserved bi-allelic *RNU6ATAC* variants are associated with an excess of significant minor intron retention (*θ*) outliers (A) Outlier individual A1 with an excess of minor intron retention (*θ*) outliers. Plot showing the number of significant (*q* < 0.05, abs(Δ*Ψ*) ≥ 0.3) minor intron retention (*θ*) outliers in our 385-person whole-blood cohort. Each dot represents an individual, and the *y* axis position represents the number of significant minor intron retention (*θ*) outliers detected in that individual. The *x* axis is ordered by the number of significant minor intron retention (*θ*) outliers per individual. The red, labeled circle represents individual A1 with rare, bi-allelic variants in *RNU6ATAC*, while the yellow circles represent individuals with RNU4atac-opathy. (B) Outlier individual B1 with an excess of minor intron retention (*θ*) outliers. Plot showing the number of significant (*q* < 0.05, abs(Δ*Ψ*) ≥ 0.3) minor intron retention (*θ*) outliers in our 139-person fibroblast cohort. Each dot represents an individual, and the *y* axis position represents the number of significant minor intron retention (*θ*) outliers detected in that individual. The *x* axis is ordered by the number of significant minor intron retention (*θ*) outliers per individual. The red, labeled circle represents individual B1 with rare, bi-allelic variants in *RNU6ATAC*. (C) Conservation of the *RNU6ATAC* genomic region across vertebrates. Nucleotide-level conservation scores for the *RNU6ATAC* gene were computed using PhyloP across 100 vertebrate species. Representative aligned sequences from human, rhesus macaque, mouse, dog, elephant, chicken, *Xenopus tropicalis*, and zebrafish are shown. The two variants identified in individual A1 are marked by purple solid and dashed arrows, highlighting their position within highly conserved regions. The two variants identified in individual B1 are marked by teal solid and dashed arrows, highlighting their position within highly conserved regions. The variant found in individual C1 is indicated by a dark red arrow, showing its location within highly conserved regions. (D) Disruption of U4atac-U6atac RNA duplex involved in minor spliceosome catalysis. A secondary structure model of the RNA duplex formed between U4atac and U6atac is necessary for creating the catalytic core of the minor spliceosome. The two variants identified in individual A1 are marked with purple solid and dashed arrows. The two variants identified in individual B1 are marked with teal solid and dashed arrows. The variant found in individual C1 is indicated by a dark red arrow. Nucleotide positions in U4atac/U6atac bimolecule are colored according to their functional importance in minor splicing, as the legend indicates.

To better reflect the molecular etiology of minor spliceopathies, we refined this method to quantify retention specifically in minor (U12-type) introns, rather than all introns within MIGs. When applied to the original 385-sample whole-blood cohort, our refined method recapitulated the same five outliers. These individuals exhibited a profound excess of MIR (mean: 288.8, median: 268 events, IQR: 254–309) compared with the remainder of the cohort (mean: 0.1, median: 0, IQR: 0–0; Figure 1A). Specifically, individual A1 showed a distinct outlier profile (*Z* score: 7.7), harboring 254 MIR outliers across 142 MIGs (Figure 1A). Importantly, focusing specifically on minor introns significantly reduced background noise, and the rate of retention in unaffected samples dropped from a mean of 1.7 events in the original analysis to 0.1 events in our refined analysis.

We subsequently extended this framework to a fibroblast cohort of 139 individuals from the Stanford UDN clinical site and the GREGoR consortium. This analysis identified a single additional outlier, individual B1, for whom fibroblasts were the only available tissue sample. This individual exhibited an excess of MIR outliers (*Z* score: 10.4). Individual B1 had 16 MIR events in 14 MIGs, whereas the rest of the cohort had, on average, less than one MIR event (mean: 0.2, median: 0, IQR: 0–0) in less than one MIG (mean: 0.2, median: 0, IQR: 0–0) (Figure 1B).

### Genome reanalysis

We identified bi-allelic *RNU6ATAC* (GenBank: NR_023344.1) variants in all three affected individuals, consistent with autosomal recessive inheritance (Table 1). In the discovery cohort, individual A1 was compound heterozygous for variants affecting the U4atac/U6atac stem I region^5^ (NR_023344.1: n.28C>T, maternal inheritance) and the U4atac/U6atac stem II region^5^ (NR_023344.1:n.36T>G, paternal inheritance). Individual B1 harbored bi-allelic variants disrupting the U4atac/U6atac stem I/II boundary (NR_023344.1: n.30C>T, maternal inheritance), which was previously reported in ClinVar^19^ as of uncertain significance (ClinVar: RCV004764396.1), and the Central Stem-loop (NR_023344.1: n.64C>G, paternal inheritance), a critical binding site for the splicing factor CENATAC^20^ (centrosomal AT-AC splicing factor [MIM: 6200142]).

**Table 1:** Summary of genetic findings for both affected individuals

Feature	Individual A1		Individual B1		Individual C1	
	Compound heterozygous		Compound heterozygous		Homozygous	
Genomic coordinate (hg38)	9-134164537-G-A	9-134164529-A-C	9-134164535-G-A	9-134164501-G-C	9-134164522-C-T	9-134164522-C-T
HGVS.C (NR_023344.1)	n.28C>T	n.36T>G	n.30C>T	n.64C>G	n.43G>A	n.43G>A
Inheritance	maternal	paternal	maternal	paternal	maternal	paternal
gnomAD version 4.1.0 allele frequency	0.0000789	absent	0.00000658	absent	0.00001313	0.00001313
gnomAD version 4.1.0 homozygotes	0	NA	0	NA	0	0
CADD score	21	18	21	19	19	19
PhyloP100 score	9.55	7.12	7.62	3.98	4.72	4.72
ClinVar classification	VUS	VUS	VUS	VUS	VUS	VUS
ClinVar accession number	SCV007299091	SCV007299092	SCV007299093	SCV007299094	SCV007334818	SCV007334818
snRNA region affected	U6atac/U4atac stem I pairing region	U6atac/U4atac stem II pairing region	stem I/II boundary of the U4atac/U6atac bimolecule	central stem-loop region	U6atac/U4atac stem II pairing region	U6atac/U4atac stem II pairing region
RNA sequencing sample type	whole blood		fibroblasts		NA	
Transcriptomic *θ* outliers	254 minor intron retention outliers		16 minor intron retention outliers		NA	

All identified variants are rare and affect evolutionarily conserved positions. Computational predictions support their potential deleterious effects. The table includes ClinVar classifications, ClinVar accession numbers, and transcriptomic outlier status. CADD, combined annotation-dependent depletion; NA, not applicable; snRNA, small nuclear RNA; VUS, variant of uncertain significance.

In the replication cohort, individual C1 was homozygous for a U4atac/U6atac stem II region variant (NR_023344.1: n.43G>A),^10^ with both parents genotyped as carriers. Structurally, n.43 base pairs with *RNU4ATAC* position n.8, a recognized mutational hotspot,^10^ indicating that disrupting this specific interaction destabilizes the minor spliceosome complex. Detailed genetic findings are included in supplemental material 2.

All identified variants are absent or extremely rare in gnomAD version 4.1.0,^17^^,^^18^ and according to PhyloP100,^16^ they impact highly conserved nucleotides predicted to be essential for minor spliceosome assembly and function (Figures 1C and 1D).

Notably, all *RNU6ATAC* variants identified in this study yielded combined annotation-dependent depletion (CADD^21^) Phred scores between 18 and 21, well above the region-specific threshold of 11.44 set by Tenywa et al. for pathogenic non-coding RNAs (ncRNAs).^22^

To identify additional affected individuals, we submitted the *RNU6ATAC* gene to GeneMatcher,^23^ which yielded one non-informative research-interest match, and engaged international collaborators, leading to the inclusion of individual C1.

### Clinical overview of the study cohort

**Figure 2:**
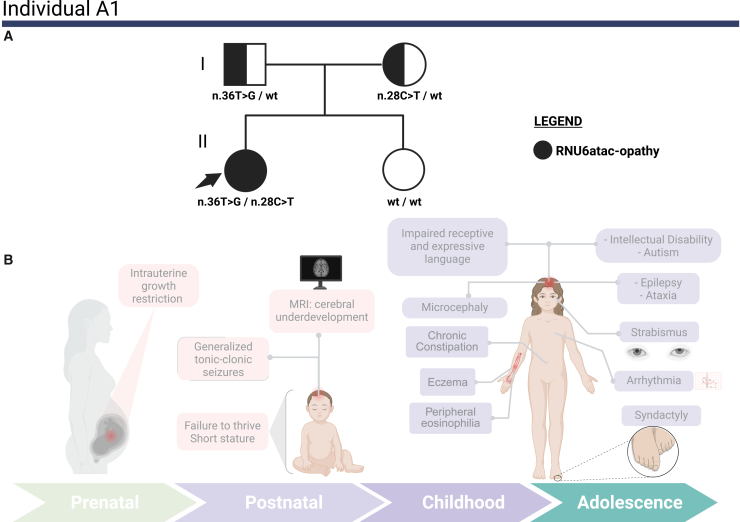
Pedigree and clinical features of individual A1, with compound heterozygous *RNU6ATAC* variants (A) Pedigree of individual A1’s family: the pedigree illustrates the segregation pattern of *RNU6ATAC* variants (NR_023344.1: n.36T>G and NR_023344.1: n.28C>T) in a compound heterozygous state in the proband (individual A1), who is marked with an arrow. The proband is represented by a fully black circle, indicating that she is affected by RNU6atac-opathy. The father is shown as a square half-filled in black, indicating carrier status for the NR_023344.1: n.36T>G variant. The mother is depicted as a circle half-filled in black, showing carrier status for the NR_023344.1: n.28C>T variant. The sister of the proband is represented by a white circle, consistent with a wild-type genotype for both variants, confirming she does not carry either variant. Segregation was confirmed through orthogonal Sanger sequencing. (B) Illustration of key clinical features: a schematic representation of individual A1’s major clinical manifestations across different life stages, from prenatal to her current age of 14 years.

**Figure 3:**
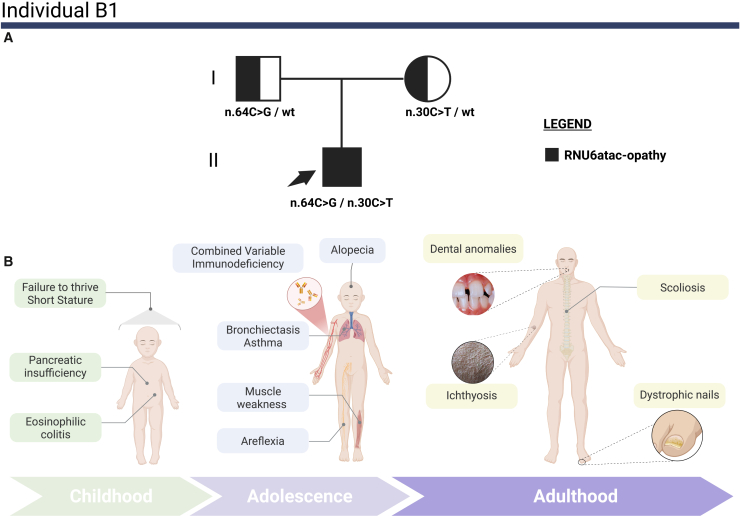
Pedigree and clinical features of individual B1 with compound heterozygous *RNU6ATAC* variants (A) Pedigree of individual B1’s family: the pedigree illustrates the inheritance pattern of *RNU6ATAC* variants (NR_023344.1: n.30C>T and NR_023344.1: n.64C>G) in a compound heterozygous state in the proband (individual B1), who is indicated by an arrow. The proband is represented by a fully black circle, indicating that he is affected by RNU6atac-opathy. The father is shown as a square half-filled in black, indicating carrier status for the NR_023344.1: n.64C>G variant. The mother is depicted as a circle half-filled in black, indicating carrier status for the NR_023344.1: n.30C>T variant. Segregation was confirmed through orthogonal Sanger sequencing. (B) Illustration of key clinical features: a schematic representation of individual B1’s major clinical manifestations across different life stages, from childhood through adolescence to adulthood.

**Figure 4:**
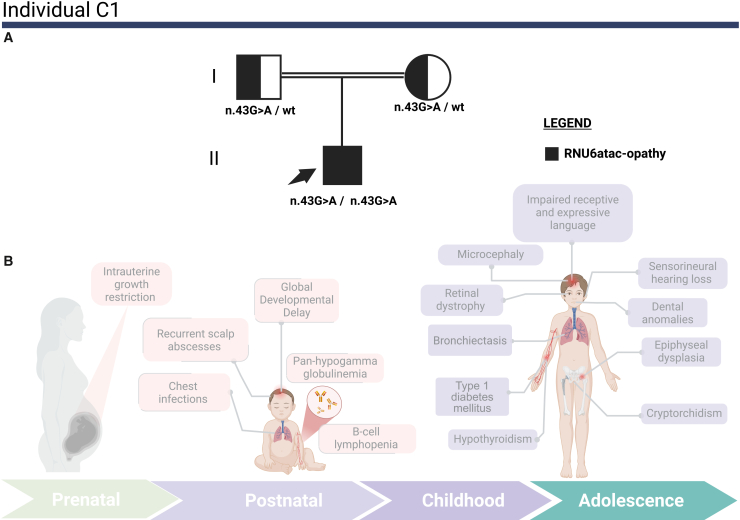
Pedigree and clinical features of individual C1 with a homozygous *RNU6ATAC* variant (A) Pedigree of individual C1’s family: the pedigree illustrates the inheritance pattern of *RNU6ATAC* variants (NR_023344.1: n.43G>A) in a homozygous state in the proband (individual C1), who is indicated by an arrow. The proband is represented by a fully black circle, indicating that he is affected by RNU6atac-opathy. The father is shown as a square half-filled in black, indicating carrier status for the NR_023344.1: n.43G>A variant. The mother is depicted as a circle half-filled in black, indicating carrier status for the NR_023344.1: n.43G>A variant. Segregation was confirmed through orthogonal Sanger sequencing. (B) Illustration of key clinical features: a schematic representation of individual C1’s major clinical manifestations across different life stages, from childhood through adolescence.

The discovery cohort comprises two individuals with distinct multisystem presentations. Individual A1 is a 14-year-old female with intrauterine growth restriction, microcephaly, refractory epilepsy, and cerebral structural anomalies. Her course is further complicated by ataxia, autism, severe intellectual disability, and marked peripheral eosinophilia (Figures 2A and 2B). Individual B1 is a 30-year-old male with a multisystem disorder characterized by prominent immune dysfunction, endocrinopathy, and ectodermal abnormalities. His clinical history includes primary hypothyroidism, failure to thrive, bronchiectasis, alopecia universalis, chronic inflammatory demyelinating polyneuropathy, and combined variable immunodeficiency (CVID), without neurodevelopmental involvement (Figures 3A and 3B). The replication cohort includes individual C1, a 17-year-old male who presents with microcephaly, growth failure, developmental delay, immunodeficiency, and severe skeletal dysplasia (Figures 4A and 4B). Comprehensive clinical descriptions are provided in supplemental material 2.

## Discussion

We report three unrelated individuals with bi-allelic variants in *RNU6ATAC*, the gene encoding the U6atac snRNA of the minor spliceosome.^1^ These cases expand the spectrum of phenotypes associated with defects in the minor spliceosome. Notably, the clinical features observed across these three individuals span many organ systems and resemble the pleiotropic presentation of known minor spliceopathies.^8^^,^^9^^,^^24^ Typical findings in RNU4atac-opathies (MOPD1, Roifman, and Lowry-Wood syndromes)^8^^,^^10^^,^^25^ include severe growth restriction, microcephaly, skeletal dysplasia, and cognitive impairment, with variable additional involvement of the brain, immune system, cardiovascular system, eyes, skin, gastrointestinal tract, hearing, and endocrine organs,^8^^,^^10^^,^^25^ while individuals with *RNU12* variants present with SCAR33^9^ or CDAGS syndrome.^12^

The phenotype of individual A1 aligns with a severe, neurologically focused spectrum; she has microcephaly, profound developmental impairment with intractable epilepsy, and structural brain anomalies. Her short stature also aligns with the growth characteristic of RNU4atac-opathies.^8^^,^^10^^,^^25^ The presence of ataxia mirrors the presentation of SCAR33.^9^ Immune dysfunction is not prominent in A1; she did not have recurrent infections, but her hypereosinophilia suggests immune dysregulation.

In contrast, the phenotype of individual B1 lacked microcephaly, epilepsy, and cognitive impairment, but was defined by a predominantly systemic presentation featuring a profound CVID-like immunodeficiency (supplemental material 3). Beyond the shared clinical features of growth failure and eosinophil-related inflammation, the immunophenotype of individual B1 mirrors characteristics seen in RNU4atac-opathy,^26^ especially severe B cell lymphopenia and hypogammaglobulinemia consistent with the B cell developmental block described by Heremans et al.^26^ However, the immune profile of individual B1 is distinguished by significant T cell pathology not typically seen in Roifman syndrome,^26^ including CD4^+^ lymphopenia. Along with his autoimmune polyendocrinopathy-candidiasis-ectodermal dystrophy-like ectodermal features, we hypothesize that *RNU6ATAC* dysfunction might perturb MIGs regulating lymphocyte development more broadly, likely impairing both bone marrow B cell formation^26^ and thymic T cell maturation checkpoints.

Importantly, identifying individual C1 fills a crucial gap in this phenotypic spectrum. Individual C1 exhibits a “bridging phenotype,” which combines the constitutional growth failure and developmental delay of A1 with the severe immunodeficiency (pan-hypogammaglobulinemia, bronchiectasis) and autoimmunity (type 1 diabetes) observed in individual B1. This unification suggests that the neurodevelopmental and immune-skeletal presentations are not separate entities but rather endpoints of a continuous RNU6ATAC-opathy spectrum. We also emphasize the importance of ongoing identification of affected individuals and thorough molecular characterization to fully understand the genotype-phenotype correlations within this locus.

The *RNU6ATAC* variants identified in our cohort yielded CADD^21^ Phred scores of 18–21, well above the 11.44 threshold proposed by Tenywa et al. for prioritizing deleterious variants in ncRNA regions.^22^ This range is consistent with the recurrent pathogenic *RNU4-2* insertion (CADD 20.8), supporting the notion that region-aware *in silico* scores can effectively flag deleterious changes in spliceosomal snRNAs.^22^ Additionally, all five *RNU6ATAC* variants identified in the cohort map to conserved regions of the U6atac snRNA secondary structure, providing clues to how they may disrupt the minor spliceosome. The bi-allelic variants in A1 (NR_023344.1: n.28C>T and NR_023344.1: n.36T>G) likely impair the formation of the U4atac/U6atac di-snRNP complex by disrupting key base-pairing interactions in stem I and stem II.^3^^,^^20^ Similarly, individual B1 harbored two variants (NR_023344.1: n.30C>T and NR_023344.1: n.64C>G) outside the canonical stem I and stem II domains. These positions may subtly destabilize RNA structure or disrupt interactions with accessory proteins such as CENATAC,^20^ likely leading to qualitatively different effects on minor spliceosome function. Notably, individual C1 is homozygous for a variant in the U4atac/U6atac stem II region (NR_023344.1: n.43G>A) that base pairs directly with nucleotide n.8 of *RNU4ATAC*.^10^ Since *RNU4ATAC* n.8 is a known pathogenic hotspot reported in multiple affected individuals with RNU4ATAC-opathy,^10^ this provides structural evidence that disrupting this specific intermolecular interaction could interfere with the normal function of the minor spliceosome.

Transcriptome-wide analysis supports the hypothesis that the splicing defect in individuals A1 and B1 is specific to MIR, paralleling the selective splicing anomalies reported in RNU4atac-opathy.^5^ This conserved transcriptomic pattern indicates a common mechanism of minor spliceopathies, providing molecular evidence that RNU6atac-opathy belongs in the expanding group of minor spliceopathies. In our study, the markedly higher excess of MIR outliers observed in whole blood (individual A1, 252 events) compared with fibroblasts (individual B1, 16 events) may reflect a convergence of tissue-specific transcript abundance and differential biological sensitivity, but we cannot discount intrinsic differences in the degree of impact of the variants.^14^^,^^27^ Previous evidence shows that the transcriptional landscape varies by tissue; many MIGs are expressed at low levels in fibroblasts, which limits the pool of detectable transcripts for intron retention analysis relative to blood.^27^ We acknowledge that a direct comparison would utilize paired samples; however, material was limited to blood for A1 and fibroblasts for B1. Our findings parallel those in RNU4atac-opathy,^5^^,^^27^ in which hematopoietic lineages exhibit more severe U12-type intron mis-splicing than mesenchymal cells.^27^ This aligns with the concept that the low-abundance minor spliceosome might create a bottleneck in rapidly dividing tissues.^14^ Collectively, these data likely support a model in which *RNU6ATAC* variants reduce the functional reserve of the minor spliceosome below the threshold required for hematopoietic homeostasis, while remaining partially sufficient for basal splicing in fibroblasts.^27^ More affected individuals will need to be identified to better understand genotype-phenotype correlations in this condition.

Finally, our findings underscore the necessity of WGS to detect variants in RNU6ATAC, which are missed by standard WES. Furthermore, we reveal a significant gap in rare disease transcriptomics. Since minor introns account for less than 0.5% of all introns in the human genome,^1^ standard global retention metrics often overlook specific minor spliceosome dysfunctions. To capture this signal, we recommend that clinical bioinformatics pipelines incorporate targeted filtering that intersects splicing outliers with minor intron annotations.^1^ We advocate adopting the FRASER-based^15^ outlier detection framework used in Arriaga et al.^5^ and refined in this study, as it effectively detects MIR outliers that would otherwise remain overlooked by standard global analytic tools.^5^

### Conclusion

We identified bi-allelic *RNU6ATAC* variants in three individuals, guided by a distinctive transcriptomic signature of MIR in the discovery cohort and validated in an independent replication cohort. This supports *RNU6ATAC* as a disease-associated gene, defining a multisystem minor spliceopathy. Our study demonstrates that integrating transcriptomic signatures and genomic analysis offers a valuable diagnostic tool for variant pathogenicity. Ultimately, further interdisciplinary studies will be essential to fully elucidate the mechanisms of RNU6atac-opathy.

## Data and code availability

All the variants in *RNU6ATAC* identified in this study were submitted to ClinVar (https://www.ncbi.nlm.nih.gov/clinvar/) (GenBank: NR_023344.2) (submitter ID 505999, UDN; submitter ID 510380, Lifera Omics). The ClinVar accession numbers of each variant are listed in Table S1.

All RNA-seq data for samples enrolled in GREGoR are available in AnVIL through dbGaP (phs003047.v3.p2). Most RNA-seq data for samples enrolled in the UDN are available through dbGaP (phs001232.v7.p3). The remaining data will be uploaded to dbGaP as a part of the next UDN data freeze. Data are also available before the next freeze by a request to the corresponding authors with evidence of dbGaP approval for UDN data.

## Consortia

Members of the UDN: Alyssa A. Tran, Arjun Tarakad, Ashok Balasubramanyam, Brendan H. Lee, Carlos A. Bacino, Daryl A. Scott, Elaine Seto, Gary D. Clark, Hongzheng Dai, Hsiao-Tuan Chao, Ivan Chinn, James P. Orengo, Jill A. Rosenfeld, Kim Worley, Lindsay C. Burrage, Lisa T. Emrick, Lorraine Potocki, Monika Weisz Hubshman, Richard A. Lewis, Ronit Marom, Seema R. Lalani, Shamika Ketkar, Tiphanie P. Vogel, William J. Craigen, Jared Sninsky, Lauren Blieden, Sandesh Nagamani, Hugo J. Bellen, Michael F. Wangler, Oguz Kanca, Shinya Yamamoto, Christine M. Eng, Patricia A. Ward, Pengfei Liu, Adeline Vanderver, Cara Skraban, Edward Behrens, Gonench Kilich, Kathleen Sullivan, Kelly Hassey, Ramakrishnan Rajagopalan, Rebecca Ganetzky, Vishnu Cuddapah, Anna Raper, Daniel J. Rader, Giorgio Sirugo, Anne Slavotinek, Christopher Mayhew, Eneida Mendonca, Ziyuan Guo, Allyn McConkie-Rosell, Kelly Schoch, Mohamad Mikati, Nicole M. Walley, Rebecca C. Spillmann, Vandana Shashi, Alan H. Beggs, Calum A. MacRae, David A. Sweetser, Deepak A. Rao, Edwin K. Silverman, Elizabeth L. Fieg, Frances High, Gerard T. Berry, Ingrid A. Holm, J. Carl Pallais, Joan M. Stoler, Joseph Loscalzo, Lance H. Rodan, Laurel A. Cobban, Lauren C. Briere, Matthew Coggins, Melissa Walker, Richard L. Maas, Susan Korrick, Jessica Douglas, Cecilia Esteves, Emily Glanton, Isaac S. Kohane, Kimberly LeBlanc, Shamil R. Sunyaev, Shilpa N. Kobren, Brett H. Graham, Erin Conboy, Francesco Vetrini, Kayla M. Treat, Khurram Liaqat, Lili Mantcheva, Stephanie M. Ware, Kathleen Page, Paul Auwaerter, Yuka Manabe, Carlos A. Pardo-Villamizar, Julie Hoover-Fong, Philip Dane Witmer, Winston Timp, Matthew Robinson, Zackary Dov Berger, Elizabeth Wohler, Nara Sobreira, Arian Nouraee, Carlos Prada, Erica Davis, Kai Lee Yap, Kelly Regan-Fendt, María Paula Silva, Patrick McMullen, Breanna Mitchell, Brendan C. Lanpher, Devin Oglesbee, Eric Klee, Filippo Pinto e Vairo, Ian R. Lanza, Kahlen Darr, Lindsay Mulvihill, Lisa Schimmenti, Queenie Tan, Surendra Dasari, Abdul Elkadri, Brett Bordini, Donald Basel, James Verbsky, Julie McCarrier, Michael Muriello, Michael T. Zimmermann, Adriana Rebelo, Carson A. Smith, Deborah Barbouth, Guney Bademci, Joanna M. Gonzalez, Kumarie Latchman, LéShon Peart, Mustafa Tekin, Nicholas Borja, Stephan Zuchner, Stephanie Bivona, Willa Thorson, Herman Taylor, Rakale C. Quarells, Ayuko Iverson, Bruce Gelb, Charlotte Cunningham-Rundles, Eric Gayle, Joanna Jen, Louise Bier, Mafalda Barbosa, Manisha Balwani, Mariya Shadrina, Rachel Evard, Saskia Shuman, Susan Shin, Vaidehi Jobanputra, Andrea Gropman, Barbara N. Pusey Swerdzewski, Camilo Toro, Colleen E. Wahl, Donna Novacic, Ellen F. Macnamara, John J. Mulvihill, Maria T. Acosta, Precilla D’Souza, Valerie V. Maduro, Ben Afzali, Ben Solomon, Cynthia J. Tifft, David R. Adams, Elizabeth A. Burke, Francis Rossignol, Heidi Wood, Jiayu Fu, Joie Davis, Leoyklang Petcharet, Lynne A. Wolfe, Margaret Delgado, Marie Morimoto, Marla Sabaii, MayChristine V. Malicdan, Neil Hanchard, Orpa Jean-Marie, Wendy Introne, William A. Gahl, Yan Huang, Andrew Stergachis, Danny E. Miller, Elisabeth Rosenthal, Elizabeth Blue, Elsa Balton, Emily Shelkowitz, Eric Allenspach, Fuki M. Hisama, Gail P. Jarvik, Ghayda Mirzaa, Ian Glass, Kathleen A. Leppig, Katrina Dipple, Mark Wener, Martha Horike-Pyne, Michael Bamshad, Peter Byers, Runjun Kumar, Seth Perlman, Sirisak Chanprasert, Virginia Sybert, Wendy Raskind, Nitsuh K. Dargie, Chun-Hung Chan, Dr. Francisco Bustos Velasq, Isum Ward, Jason Schend, Jennifer Morgan, Megan Bell, Miranda Leitheiser, Mohamad Saifeddine, Paul Berger, Rachel Li, Taylor Beagle, Alexander Miller, Beatriz Anguiano, Beth A. Martin, Brianna Tucker, Chloe M. Reuter, Devon Bonner, Elijah Kravets, Hector Rodrigo Mendez, Holly K. Tabor, Jacinda B. Sampson, Jason Hom, Jennefer N. Kohler, Jennifer Schymick, John E. Gorzynski, Jonathan A. Bernstein, Kevin S. Smith, Laura Keehan, Laurens Wiel, Matthew T. Wheeler, Meghan C. Halley, Mia Levanto, Page C. Goddard, Paul G. Fisher, Rachel A. Ungar, Raquel L. Alvarez, Sara Emami, Shruti Marwaha, Stephen B. Montgomery, Suha Bachir, Tanner D. Jensen, Taylor Maurer, Terra R. Coakley, Euan A. Ashley, Anna Hurst, Brandon M. Wilk, Bruce Korf, Elizabeth A. Worthey, Kaitlin Callaway, Martin Rodriguez, Pongtawat Lertwilaiwittaya, Reaford Blackburn, Tammi Skelton, Tarun K.K. Mamidi, Teneasha Washington, Andrew B. Crouse, Jordan Whitlock, Mariko Nakano-Okuno, Matthew Might, William E. Byrd, Albert R. La Spada, Changrui Xiao, Elizabeth C. Chao, Eric Vilain, Jose Abdenur, Kirsten Blanco, Maija-Rikka Steenari, Rebekah Barrick, Richard Chang, Sanaz Attaripour, Suzanne Sandmeyer, Tahseen Mozaffar, Alden Huang, Andres Vargas, Bianca E. Russell, Brent L. Fogel, Esteban C. Dell’Angelica, George Carvalho, Julian A. Martínez-Agosto, Layal F. Abi Farraj, Manish J. Butte, Martin G. Martin, Naghmeh Dorrani, Neil H. Parker, Rosario I. Corona, Stanley F. Nelson, Yigit Karasozen, Dana Sayer, Jennifer Tousseau, Aaron Quinlan, Alistair Ward, Ashley Andrews, Corrine K. Welt, Dave Viskochil, Erin E. Baldwin, John Carey, Justin Alvey, Lorenzo Botto, Nicola Longo, Paolo Moretti, Rebecca Overbury, Russell Butterfield, Steven Boyden, Thomas J. Nicholas, Matt Velinder, Gabor Marth, Pinar Bayrak-Toydemir, Rong Mao, Monte Westerfield, John A. Phillips III, Kimberly Ezell, Lynette Rives, Rizwan Hamid, Alyson Krokosky, Ashley McMinn, Cathy Shyr, Eric Gamazon, Joy D. Cogan, Lakshitha Perera, Lisa Bastarache, Mary Koziura, Thomas Cassini, Alex Paul, Dana Kiley, Daniel Wegner, Erin McRoy, Jennifer Wambach, Kathy Sisco, Patricia Dickson, F. Sessions Cole, Dustin Baldridge, Jimann Shin, Lilianna Solnica-Krezel, Stephen C. Pak, Timothy Schedl, Allen Bale, Carol Oladele, Caroline Hendry, Emily Wang, Hua Xu, Hui Zhang, Lauren Jeffries, María José Ortuño Romero, Mark Gerstein, Michele Spencer-Manzon, Monkol Lek, Nada Derar, Odelya Kaufman, Shrikant Mane, Teodoro Jerves Serrano, Vasilis Vasiliou, Winston Halstead, and Yong-Hui Jiang.

Members of the GREGoR consortium: Siwaar Abouhala, Sophia Adelson, Kaileigh Ahlquist, Miguel Almalvez, Emily Alsentzer, Raquel Alvarez, Mutaz Amin, Peter E. Anderson, Kailyn Anderson, Euan Ashley, Themistocles Assimes, Light Auriga, Christina Austin-Tse, Michael J. Bamshad, Rebekah Barrick, Samantha Baxter, Sairam Behera, Shaghayegh Beheshti, Gill Bejerano, Sami Belhadj, Seth Berger, Jon Bernstein, Sabrina Best, Kirsten Blanco, Benjamin Blankenmeister, Elizabeth E. Blue, Krista Bluske, Eric Boerwinkle, Emily Bonkowski, Devon Bonner, Philip M. Boone, Leandros Boukas, Denver Bradley, Harrison Brand, Kati J. Buckingham, Daniel Calame, Colleen Carlston, Jennefer Carter, Silvia Casadei, Lisa Chadwick, Clarisa Chavez, Ziwei Chen, Allison Cheney, Yong-Han Cheng, Ivan Chinn, Jessica X. Chong, Zeynep Coban-Akdemir, Andrea J. Cohen, Sarah Conner, Matthew P. Conomos, Karen Coveler, Laura Covill, Allen Cui Ya, Colleen P. Davis, Moez Dawood, Ivan de Dios, Celine de Esch, Emmanuèle Délot, Wei Deng, Salil Deshpande, Stephanie DiTroia, Harsha Doddapaneni, Haowei Du, Michael Duyzend, Michael Duyzend, Iman Egab, Evan E. Eichler, Sara Emami, Ivy Evergreen, Mira Gandhi, Vijay Ganesh, Brandon Garcia, Kiran Garimella, Richard Gibbs, Sophia B. Gibson, Casey Gifford, Carmen Glaze, Pagé Goddard, Stephanie Gogarten, Nikhita Gogate, William W. Gordon, John E. Gorzynski, William Greenleaf, Christopher Grochowski, Emily Groopman, Rodrigo Guarischi-Sousa, Sanna Gudmundsson, (A. Gus) Gustafson Jonas, Stacey Hall, Caitlin Harrington, John Harting, William T. Harvey, Sohaib Hassan, Megan Hawley, Benjamin D. Heavner, Martha Horike-Pyne, Yun-Hua Hsiao, Jianhong Hu, Yongqing Huang, Karan Jaisingh, Minal Jamsandekar, Gail P. Jarvik, Tanner Jensen, Shalini Jhangiani, David Jimenez-Morales, Christopher Jin, Aimee Juan, Ahmed K. Saad, Jessica Kain, Rachid Karam, Laura Keehan, Sky Kim, Hadley King Charles, Julia Klugherz, Arthur Ko, Anshul Kundaje, Soumya Kundu, Samuel M. Lancaster, Katie Larsson, Arthur Lee, Gabrielle Lemire, Mia Levanto, Jesse Levine, Wei Li, Pengfei Liu, Bojan Losic, Jonathan LoTempio, James (Jim) Lupski, Jialan Ma, Daniel MacArthur, Annelise Y. Mah-Som, Medhat Mahmoud, Brian Mangilog, Dana Marafi, Daniel Marten, Eva Martinez, Colby T. Marvin, Shruti Marwaha, F. Kumara Mastrorosa, Dena Matalon, Taylor Maurer, Susanne May, Sean R. McGee, Lauren Meador, Heather C Mefford., Rodrigo Mendez Hector, Olfa Messaoud, Alexander Miller, Danny E. Miller, Romal Mitr, Stephen Montgomery, Yulia Mostovoy, Mariana Moyses, Chloe Munderloh, Donna Muzny, Ashana Neale, Sarah C. Nelson, Matthew B. Neu, Jonathan Nguyen, Thuy-mi P. Nguyen, Annie Niehaus, Robert Nussbaum, Emily O’Heir, Briana O’Leary, Melanie O’Leary, Sebastian Ochoa Gonzalez, Jeren Olsen, Osei-Owusu Ikeoluwa, Anne O’Donnell-Luria, Miranda P.G. Zalusky, Evin Padhi, Lynn Pais, Piyush Panchal, Shruti Pande, Karynne E. Patterson, Sheryl Payne, Davut Pehlivan, Paul Petrowski, Alicia Pham, Georgia Pitsava, Astaria (Sara) Podesta, Elizabeth Porter, Jennifer Posey, Jaime Prosser, Guanghao Qi, Wanqiong Qiao, Thomas Quertermous, Archana Rai, Heidi Rehm, Chloe Reuter, Matthew A. Richardson, Andres Rivera-Munoz, Lindsay Romo, Oriane Rubio, Kathryn Russell, Aniko Sabo, Ismail Safi, Monica Salani, Kaitlin Samocha, Armando Sanchez-Conde, Alba Sanchis-Juan, Sarah Savage, Jacob Schmidt, Evette Scott, Stuart Scott, Adriana E. Sedeño-Cortés, Fritz Sedlazeck, Jillian Serrano, Gulalai Shah, Deepali Shinde, Ali Shojaie, Moriel Singer-Berk, Mugdha Singh, Riya Sinha, Joshua D. Smith, Kevin Smith, Hana Snow, Michael Snyder, Kayla Socarras, Olivia M. Sommerland, Lea M. Starita, Brigitte Stark, Sarah Stenton, Andrew B. Stergachis, Adrienne Stilp, V. Reid Sutton, Elliott G. Swanson, Jui-Cheng Tai, Michael Talkowski, Christina G.Tise, Catherine C. Tong, Philip Tsao, Rachel Ungar, Grace VanNoy, Eric Vilain, Gaby Villard, Mitchell R. Vollger, Isabella Voutos, Kim Walker, Juliana Walrod, Chia-Lin Wei, Ben Weisburd, Jeffrey M. Weiss, Chris Wellington, Ziming Weng, Lauren Westerfield, Matthew Wheeler, Marsha Wheeler, Laurens Wiel, Michael Wilson, Monica Wojcik, Chee Hong Wong, Issac Wong, Quenna Wong, Frank Wong, Changrui Xiao, Jiaoyang Xu, Rachita Yadav, Yao Yang, Qian Yi, Jiye Yu, Bo Yuan, Christina Zakarian, Jimmy Zhen.

## Acknowledgments

We thank all research individuals in the UDN and GREGoR consortium. We would also like to thank members of the Wheeler lab, the Montgomery lab, the GREGoR consortium, the UDN, and the Stanford Center for Undiagnosed Diseases, who gave invaluable feedback and assistance throughout this project. Figures were created in BioRender (https://BioRender.com/emhjjfv). This work required computing resources from the Stanford Genetics Bioinformatics Service Center (supported by NIH Instrumentation grant S10 OD025082). The work would not have been possible without the Stanford SCG cluster administrators, specifically Chris Jeon and Karl Kornel. R.J.L. was supported by CNCDP-K12 funding. T.M.A. was supported by the National Science Foundation Graduate Research Fellowship under grant no. DGE-2146755. Research reported in this paper was partly funded by the 10.13039/100000002National Human Genome Research Institute at the National Institutes of Health, United States, as part of the GREGoR consortium, through grant nos. U01HG011762 and U01HG011755. This publication was also supported by the 10.13039/100000065National Institute of Neurological Disorders and Stroke of the 10.13039/100000002National Institutes of Health under award number U01NS134358. The content is solely the responsibility of the authors and does not necessarily represent the official views of the National Institutes of Health.

## Author contributions

Project conceptualization, V.S.G., A.O.L., J.A.B., S.B.M., and M.T.W.; ethics approval, D.E.B.; participant recruitment and clinical data contribution, S.E., R.J.L., D.N., R.M., D.E.B., F.S.A., A.A., B.A., and K.B.; data generation, K.S.S., S.A.S., L.L., Z.N., F.S.A., and K.B.; data analysis, T.M.A., V.S.G., J.M., R.M., R.A.U., A.M.M., J.N., S.M., and A.W.; figure generation, R.M., T.M.A., and D.E.B.; writing – original draft, R.M., T.M.A., and D.E.B.; writing – review & editing, all authors.

## Declaration of interests

S.B.M. is a member of the scientific advisory boards of MyOme, PhiTech, and Valinor Therapeutics. A.W. is chief executive officer of Frameshift Labs (developer of Mosaic). R.J.L. is an unpaid member of the scientific advisory boards for the Timothy Syndrome Foundation and the Timothy Syndrome Alliance.

## Web resources

ClinVar, https://www.ncbi.nlm.nih.gov/clinvar/
Online Mendelian Inheritance in Man (OMIM), http://www.omim.org
UCSC Genome Browser^16^ “Exome Probesets” track (GRCh38/hg38), http://genome.ucsc.edu

## Supplemental information

Document S1. Supplemental material 1–3 and Table S1

Document S2. Article plus supplemental information
