# Supplementary material for: Bi-allelic RNU6ATAC variants cause a minor spliceopathy characterized by transcriptome-wide minor intron retention and multisystem manifestations
Source: HGG Adv. 2026 Mar 9;7(2):100588. doi: 10.1016/j.xhgg.2026.100588 (PMC13049632; doi:10.1016/j.xhgg.2026.100588)
Supplement: Document S1. Supplemental material 1–3 and Table S1 [file mmc1.pdf]

HGGA, Volume 7

Supplemental information

Bi-allelic *RNU6ATAC* variants cause a minor spliceopathy characterized by transcriptome-wide minor intron retention and multisystem manifestations

Rodrigo Mendez, Taylor M. Arriaga, Jialan Ma, Devon E. Bonner, Sara Emami, Rebecca J. Levy, Afaf Alsagheir, Bader Alhaddad, Khadijah Bakur, Rachel A. Ungar, Dena R. Matalon, Alexander M. Miller, Jonathan Nguyen, Kevin S. Smith, Stuart A. Scott, Linda Liao, Zena Ng, Shruti Marwaha, Alistair Ward, Undiagnosed Diseases Network, Genomics Research to Elucidate the Genetics of Rare Diseases Consortium, Danica Novacic, Fowzan S. Alkuraya, Jonathan A. Bernstein, Vijay S. Ganesh, Anne O'Donnell-Luria, Stephen B. Montgomery, and Matthew T. Wheeler

Supplemental Material 2

Clinical Vignettes and Genetic Findings for RNU6atac-opathy

Individual A1 [NR_023344.1: n.28C>T; NR_023344.1:n.36T>G]; one case

Individual A1 is a 14-year-old female with a multisystem neurodevelopmental phenotype, an unremarkable family history, and a healthy sister (Figure 2A). Prenatally, intrauterine growth restriction was noted. Birth weight was 2.3 kg [Z = -2.03], length was 48.3 cm [Z = -0.41], and head circumference was 33 cm [Z = -1.11]. She experienced refractory epilepsy with seizures beginning on day three of life and was hospitalized in the neonatal intensive care unit until day twenty-three of life. She was noted to have bilateral 2-3 toe syndactyly. She was readmitted soon after discharge for failure to thrive and hyperbilirubinemia. Her infantile history was notable for poor feeding with global growth restriction and early onset nystagmus at 5 months old. She had a recurrence of epilepsy at 8 months old, with focal temporal status epilepticus. A brain MRI at that time revealed cerebral underdevelopment, characterized by a relatively small genu of the corpus callosum, moderate enlargement of the lateral ventricles, and mild enlargement of the third ventricle, as well as absence of the left 6th cranial nerve in the expected location. Given a clinical exam with limited but not absent left eye abduction, that cranial nerve is presumed to be in an atypical location. Since then, she has had treatment-refractory epilepsy. She walked at 2.5 years; her first word was at 2 years. Currently, her speech consists of occasional spontaneous one- to two-word utterances, with a total spontaneous vocabulary of about five words, although she exhibits echolalia and repeats long phrases. She was diagnosed with autism and severe intellectual disability. She developed episodic ataxia at age three, which became persistent by early childhood with a wide-based gait. Ophthalmological findings included strabismus, oculomotor apraxia, and hyperopic astigmatism. She has experienced heavy menstrual bleeding since menarche, with coagulation studies demonstrating a prolonged prothrombin time. Cardiovascular evaluation revealed a heart murmur that resolved in childhood (no echocardiogram was performed), with an ECG showing a sinus rhythm and nonspecific intraventricular conduction delay. Gastrointestinal issues included chronic constipation, abdominal pain, and dysmotility (Figure 2B). Clinical genetics evaluation included chromosomal

microarray analysis, chromosomal breakage analysis, mtDNA testing, trio genome sequencing, and biochemical testing (plasma amino acids, urine organic acids, acylcarnitines, very long-chain fatty acids, and carnitine). Creatine kinase was mildly elevated at 2 years (224 U/L; ref <150) but later normalized. At her last follow-up at 13 years and 7 months old, her height was 141 cm [Z = -2.77], her weight was 37.7 kg [Z = -1.45], and her head circumference was 49 cm [Z < -2.05]. She had new flexural eczema involving the knees and elbows; the skin exam showed generalized xerosis with scattered superficial healing excoriations on the back and limbs, and a few round hyperpigmented leg patches. Laboratory testing documented marked peripheral eosinophilia with an absolute eosinophil count of 10,000/μL.

Genetic findings: Individual A1 had compound heterozygous *RNU6ATAC* (NR_023344.1) variants (The NR_023344.1: n.28C>T, maternal inheritance; The NR_023344.1: n.36T>G, paternal inheritance). The n.28C>T variant (gnomAD v4[1,2] allele frequency: 7.9×10^{-5}; CADD[3]: 21; PhyloP100[4]: 9.55) affects a nucleotide that participates in base pairing within the Stem I region of U4atac that is crucial for splicing[5]. The n.36T>G variant, absent from gnomAD v4.1.0[1,2] (CADD[3]: 18; PhyloP100[4]: 7.12)[5], disrupts a conserved uridine in the U6atac stem II region, potentially destabilizing its structure[5] (Figure 1D). Sanger sequencing confirmed that the proband's sister did not carry either of the identified *RNU6ATAC* variants.

Individual B1 [NR_023344.1: n.30C>T; NR_023344.1: n.64C>G]; one case

Individual B1 was a 30-year-old male with a multisystem disorder characterized by prominent immune dysfunction, endocrinopathy, and ectodermal abnormalities. Family history was unremarkable, with no reported consanguinity (Figure 3A). He was born at term to a 32-year-old mother after an uncomplicated pregnancy and spontaneous vaginal delivery. Infancy was notable for primary hypothyroidism and failure to thrive; he had hypogammaglobulinemia and recurrent sinopulmonary infections. Immunological evaluation revealed a phenotype of Combined Variable Immunodeficiency (CVID) manifested by low serum immunoglobulins (IgA and IgG) and, crucially, severely reduced counts of CD4+ T cell and B cell subsets (detailed immunophenotyping, see **Supplemental Material 3**).

At age five, persistent gastrointestinal symptoms prompted evaluation, revealing eosinophilic colitis and exocrine pancreatic insufficiency with chronic steatorrhea and low stool elastase, which was long-term treated with pancrelipase with good response. Protein-losing enteropathy

and malabsorption were documented. Growth parameters remained below the third percentile throughout childhood and adolescence, and in adulthood, his height and weight were 165 cm [Z < -1.61] and 42.7 kg [Z < -3.89] (body mass index approximately 15.7 kg/m²), consistent with chronic growth failure. From early childhood, he had ichthyosis, dystrophic nails, and alopecia universalis. Dental anomalies included peg-shaped teeth with irregular spacing and cupping. During adolescence, episodes of acute muscle weakness and areflexia led to a diagnosis of chronic inflammatory demyelinating polyneuropathy (CIDP); scoliosis developed, and later contractures and sarcopenia. Respiratory disease evolved with asthma, recurrent pneumonias, and chest imaging identified bronchiectasis. Despite medical complexity, he achieved typical developmental milestones and completed high school; he initiated but withdrew from community college during the first year. He had no specific learning disability, although frequent illness-related absences and suboptimal adherence to thyroid replacement impacted schooling. (Figure 3B). Given this constellation of features, he initially received a clinical diagnosis of APECED (Autoimmune Polyglandular Syndrome Type 1 [MIM: 240300]); however, AIRE (autoimmune regulator [MIM: 607358]) gene sequencing was normal. Additional genetic evaluations included trio clinical genome sequencing, which was non-diagnostic. Ultimately, in the setting of bronchiectasis and neuromuscular weakness, he developed respiratory failure requiring intubation and could not be weaned; tracheostomy was declined, and death occurred at age 30 years.

Genetic findings: Individual B1 harbored two *RNU6ATAC* variants in trans, NR_023344.1: n.30C>T, inherited from the mother, and NR_023344.1: n.64C>G inherited from the father. The NR_023344.1: n.30C>T variant (gnomAD v4.1.0[1,2] allele frequency: 6.6×10^{-6}; CADD[3]: 21; PhyloP100[4]: 7.62) is located at the Stem I/II boundary of the U4atac/U6atac bimolecule, which is essential for activation of the spliceosome before catalysis and is reported in ClinVar[6] as of uncertain significance (RCV004764396.1). The NR_023344.1: n.64C>G variant, absent from gnomAD v4.1.0[1,2] (CADD[3]: 19; PhyloP100[4]: 3.98), disrupts the distinctive central Stem-loop of U6atac. The central stem-loop is known [1,2]to be stabilized by specific protein-RNA interactions with CENATAC (centrosomal AT-AC splicing factor [MIM: 6200142]), which neutralize the RNA's negative charge. Disruptions in this region could destabilize the U6atac structure, adversely affecting minor intron splicing[7].

Individual C1 [NR_023344.1: n.43G>A, homozygous]; one case

Individual C1 is a 17-year-old male born to first-cousin parents; he also has two healthy older sisters. He was born at 32 weeks' gestation via induced vaginal delivery due to decreased fetal movements and poor fetal growth. His birth weight was 1.5 kg [Z = -1.6]; other birth parameters were not available. He required neonatal intensive care for three weeks for growth support.

The participant presented at 7 months old with a history of recurrent scalp abscesses and chest infections starting at 2 months. While the complete blood count was unremarkable: WBC 9.6 × 10^9/L, hemoglobin 118 g/L, platelets 331 × 10^9/L, ANC 3.25 × 10^9/L, ALC 4.18 × 10^9/L, immunological evaluation identified profound pan-hypogammaglobulinemia. IgG was markedly reduced at 310 mg/dL (reference range: 660–1530 mg/dL), with IgA <25 mg/dL (reference: 70–400 mg/dL) and IgM <21 mg/dL (reference: 25–259 mg/dL); IgE was 22.2 UI/mL. Lymphocyte immunophenotyping revealed B-cell lymphopenia (CD19: 238 cells/µL), whereas T-cell and NK-cell counts were preserved (CD3: 2773, CD4: 2400, CD8: 515, NK: 336 cells/µL). Despite the antibody deficiency, T-cell function appeared intact, as evidenced by normal lymphocyte responses to mitogens: Phytohemagglutinin (PHA) 260,148 CPM; Concanavalin-A (Con-A) 81,255 CPM; Pokeweed 78,394 CPM; and Pooled Allogeneic Cells 36,466 CPM. Furthermore, expression of CD40 and CD40 ligand was intact, and oxidative burst testing by flow cytometry was normal (87% of PMNs reduced DHR-123 post-PMA stimulation).

He was diagnosed with antibody deficiency and commenced on immunoglobulin replacement therapy (IVIG, later subcutaneous), which he continues to receive. Despite treatment, he suffered from recurrent lower respiratory tract infections leading to progressive lung damage. Chest CT at age 9 confirmed advanced bilateral bronchiectasis with right middle lobe obstruction.

He exhibits a complex endocrine phenotype characterized by early-onset Type 1 diabetes mellitus (diagnosed at 9 months) and acquired hypothyroidism requiring levothyroxine. He also has a history of cryptorchidism requiring orchidopexy. Postnatal growth has been severely affected; he developed microcephaly and significant short stature. Growth hormone levels were normal, and a therapeutic trial of growth hormone yielded no improvement.

Skeletal survey revealed epiphyseal dysplasia. He developed progressive lower limb deformities, specifically severe bilateral genu valgum necessitating multiple surgical interventions, including hemiepiphysiodesis at age 8 and subsequent corrective osteotomies with ligament reconstruction. However, surgical outcomes were poor, with persistent angular deformity, bilateral fixed flexion

contractures, and global knee instability (including lateral patellar dislocation and 90-degree tibial external rotation). He is currently wheelchair-bound.

Global developmental delay was noted in infancy; he sat unsupported at 1 year and walked at 3 years. He exhibits severe delays in receptive and expressive language. Cognitive testing (Beery VMI) at age 7 showed a standard score of 70 (age equivalent: 55 months). Sensory evaluation revealed progressive retinal dystrophy characterized by nyctalopia, flat visual evoked potentials (VEP), and loss of outer retinal segments on Optical coherence tomography (OCT). Additionally, although hearing was normal in early childhood, reassessment at age 15 revealed bilateral high-frequency sensorineural hearing loss (mildly sloping to moderately severe). Dental anomalies include a congenitally missing lower right second premolar.

Genetic findings: Individual C1 was found to be homozygous for the *RNU6ATAC* variant NR_023344.1:n.43G>A, both parents were confirmed carriers, while his two healthy sisters were not genotyped. This variant is rare (allele frequency: 0.00001313), being absent in homozygous states in gnomAD v4.1.0[1,2] dataset. In silico analysis supports a deleterious effect (CADD[3]: 19; PhyloP100[4]: 4.72). Structurally, the n.43G>A variant maps to Stem II of the U4atac/U6atac bimolecule, a region critical for the assembly of the di-snRNP complex. The functional importance of this nucleotide is underscored by its structural partner: *RNU6ATAC* n.43 base-pairs with nucleotide n.8 of *RNU4ATAC*. The corresponding *RNU4ATAC* n.8 position is a recognized mutational hotspot[8], with multiple substitutions (n.8C>A, n.8C>T, and n.8C>G) reported as pathogenic or likely pathogenic in ClinVar[6]. This suggests that disrupting the Watson-Crick base pairing at this specific site within Stem II destabilizes the U4atac/U6atac complex, impairing minor spliceosome function.

References

1. Karczewski KJ, Francioli LC, Tiao G, Cummings BB, Alföldi J, Wang Q, et al. The mutational constraint spectrum quantified from variation in 141,456 humans. Nature [Internet]. 2020 May;581(7809):434–43. Available from: http://dx.doi.org/10.1038/s41586-020-2308-7

2. Chen S, Francioli LC, Goodrich JK, Collins RL, Kanai M, Wang Q, et al. A genomic mutational constraint map using variation in 76,156 human genomes. Nature [Internet]. 2024 Jan;625(7993):92–100. Available from: http://dx.doi.org/10.1038/s41586-023-06045-0

3. Schubach M, Maass T, Nazaretyan L, Röner S, Kircher M. CADD v1.7: using protein language models, regulatory CNNs and other nucleotide-level scores to improve genome-wide variant predictions. Nucleic Acids Res [Internet]. 2024 Jan 5;52(D1):D1143–54. Available from: http://dx.doi.org/10.1093/nar/gkad989

4. Raney BJ, Barber GP, Benet-Pagès A, Casper J, Clawson H, Cline MS, et al. The UCSC Genome Browser database: 2024 update. Nucleic Acids Res [Internet]. 2024 Jan 5;52(D1):D1082–8. Available from: http://dx.doi.org/10.1093/nar/gkad987

5. Arriaga MT, Mendez R, Ungar RA, Bonner DE, Matalon DR, Lemire G, et al. Transcriptome-wide outlier approach identifies individuals with minor spliceopathies. medRxiv [Internet]. 2025 Jan 3;2025.01.02.24318941. Available from: http://dx.doi.org/10.1101/2025.01.02.24318941

6. Landrum MJ, Lee JM, Benson M, Brown GR, Chao C, Chitipiralla S, et al. ClinVar: improving access to variant interpretations and supporting evidence. Nucleic Acids Res [Internet]. 2018 Jan 4;46(D1):D1062–7. Available from: http://dx.doi.org/10.1093/nar/gkx1153

7. Bai R, Yuan M, Zhang P, Luo T, Shi Y, Wan R. Structural basis of U12-type intron engagement by the fully assembled human minor spliceosome. Science [Internet]. 2024 Mar 15;383(6688):1245–52. Available from: http://dx.doi.org/10.1126/science.adn7272

8. Benoit-Pilven C, Besson A, Putoux A, Benetollo C, Saccaro C, Guguin J, et al. Clinical interpretation of variants identified in RNU4ATAC, a non-coding spliceosomal gene. PLoS One [Internet]. 2020 Jul 6 [cited 2025 Mar 14];15(7):e0235655. Available from: https://journals.plos.org/plosone/article?id=10.1371/journal.pone.0235655

Immunophenotype Characterization of Individual B1.

Individual B1 had significant immune dysfunction and initially received a clinical diagnosis of APECED (Autoimmune Polyglandular Syndrome Candidiasis and Ectodermal Dysplasia), also known as autoimmune polyglandular syndrome type-1 (APS1 [MIM: 240300]). However, *AIRE* (autoimmune regulator [MIM: 607358]) gene sequencing was normal, and several features did not fully align with the typical autoimmune dysfunction of APECED, including the absence of aberrant autoantibodies and candidal infections.

He did have Combined Variable Immune Deficiency (CVID) manifested as low immunoglobulins IgA and IgG. He had recurrent sinopulmonary infections, including pneumonia, requiring hospitalization and residual chronic lung sequela of a 2.2cm cavitary lesion in the left lower lobe, bilateral bronchiectasis, tree-in-bud, as well as ground-glass opacities. Sweat chloride was 35 mmol/L, then repeated at 36 mmol/L, which is intermediate. He had chronic protein-losing enteropathy along with eosinophilia on gastrointestinal biopsies, but IgE was normal. Peripheral blood eosinophils were mildly elevated at 890 K/ul (normal 40-540 K/uL). He was never septic. He had poor responses to some vaccines, including Pneumococcal, Hepatitis B, and Polio. Rubella titer was protective. Hemophilus influenzae titer was suboptimal at 0.17 ug/mL (short-term protection >0.15 ug/mL, long-term protective >1.0 ug/mL), and he subsequently developed H. influenzae pneumonia, confirmed by bronchoalveolar lavage samples. We did not find evidence of aberrant antibody production, including negative autoimmune antibodies for Lupus panel, Celiac, and thyroid autoantibodies. T cell mitogen-stimulation responses were normal, suggesting normal CD8 T Cell function, including a normal response to Candida antigen. On immune phenotyping, CD4 memory subsets and B cells were low. During adolescence, he developed muscle weakness and areflexia and was found to have a demyelinating polyneuropathy on nerve conduction testing. This was diagnosed as Chronic Inflammatory Demyelinating Polyneuropathy (CIDP). No known CIDP auto-antibodies were found. This was still presumed to be autoimmune, possibly CD4 T cell-mediated. Ultimately,

from CIDP, he developed muscle contractures and respiratory muscle weakness. He was treated with Intravenous Gammaglobulin. This did help prevent recurrent infections, but CIDP still progressed. Overall, his hypogammaglobulinemia may have been secondary to reduced B cell subsets due to poor/abnormal helper CD4 T cell stimulation. Low protein stores from chronic GI losses could have also played a role in his hypogammaglobulinemia. On peripheral blood flow, there was also a curious population of double-positive T cells (CD4+/CD8+) at 4.2% (normal <0.1%) of unclear significance, and CD4 memory cells were mildly low. We speculated that, together, these manifestations may point to a primary problem in proper CD4 T cell development in the thymus, including possibly poor interactions between thymic epithelial cells and maturing T cells, especially given the similarity of his phenotype to APECED. Further investigation of thymic function was not possible clinically.

Table: Notable immune labs

**Peripheral blood lab**	**Result**	**Normal Range**
**CBC (complete blood counts)**		
White blood cells total	6.14	4.23-9.07 K/uL
Lymphocytes total %	10.8	21.8-53.1%
Lymphocytes #	0.66	1.32-3.57 K/uL
**Lymphocyte phenotypes (primary immunodeficiency flow panel)**		
T4/T8 Ratio	1.39	1.11-5.17 Ratio
CD4 %	46.1	31.9-62.2 %
CD4 #	304	359-1565 /uL
CD8 %	33.1	11.2-34.8 %
CD8 #	218	178-853 /uL
CD4+/CD8+ %	4.2	<0.1%
CD4+/CD8+ #	28	0 /uL
CD4+/CD62L+/CD45RA- % (CD4 T central memory)	20.7	10.4-30.7 %

CD4+/CD62L+/CD45RA- #	137	162-614 /uL
CD4+/CD62L-/CD45RA- % (CD4 T peripheral memory)	1.6	2.3-15.6 %
CD4+/CD62L-/CD45RA- #	11	42-225 /uL
CD20 % (B cells)	0.3	3.0-19.0 %
CD20 #	2	59-329 /uL
CD19 %	0.6	3.3-19.3 %
CD19 #	4	61-321 /uL
Other subsets normal or not substantially skewed:		
NK		
NK/T		
DNT (double negative T)		
CD4 central naive		
**Immunoglobulins**		
IgG (pre-IV Ig treatment)	322.0	700-1600 mg/dL
IgA	45.0	70-400 mg/dL
IgM	48.0	40-230 mg/dL
IgE	2.4	0.0-90.0 IU/mL

Genome Sequencing

Whole Genome Sequencing (WGS) for participants A1 and B1, as well as their respective family members, was performed by Baylor Genetics, through the Undiagnosed Disease Network (UDN), using methods previously described by Splinter et al., 2018[1]. In brief, libraries were prepared using a PCR-free 550-bp insert size protocol by the Hyper Prep kit. Sequencing was performed using the Illumina NovaSeq 6000 platform for 150 bp paired-end reads. The SNPTrace Panel from the Fluidigm SNPtype platform was applied as a quality control measure. The Illumina Dragen BioIT Platform performed data analysis and interpretation. The FASTQ data were aligned to the human reference genome build GRCh38 using the Illumina Dragen BioIT Platform. Additionally, we used the Illumina Dragen haplotype-based variant calling system to perform variant calling on all resulting BAM files.

Clinical Genome Sequencing (CGS) was performed for individual C1 and his parents at Centogene. Genomic DNA was extracted from blood samples (CentoCards) using the QIASymphony magnetic bead–based method (Qiagen). DNA was fragmented by sonication, ligated to Illumina adapters, and sequenced on the Illumina HiSeq X platform, achieving >30X average genome coverage. Sequence data were aligned to the hg19 (GRCh37) reference genome. Variants were called and annotated using a validated in-house pipeline. SNVs and small indels were filtered using a semi-automated strategy, and variants with insufficient quality scores were confirmed by Sanger sequencing.

RNA-seq library preparation and sequencing

We performed RNA-sequencing on 422 whole blood samples and 139 fibroblast samples from the Genomics Research to Elucidate the Genetics of Rare diseases (GREGoR) and UDN consortia, as well as the Broad Center for Mendelian Genomics (Broad CMG) and self-funded individuals. All 422 whole blood samples were previously published in Arriaga et al., 2025[2], 287

of which were also published in Ungar et al., 2024[3]. The fibroblast cohort included samples previously published by Cummings et al., 2017[4]. Ethical and research approvals were provided by the Stanford University IRB (protocol 60837) and the National Human Genome Research Institute Institutional Review Board (IRB) (protocol 15-HG-0130, protocol 2013P001477). All participants provided informed consent.

The experimental protocol and computational pipeline for 287 of the 422 whole blood samples are described in Ungar et al., 2024[3]. Of these, eight samples were collected and processed in PAXgene tubes at the Utah UDN site before being shipped to Stanford. In brief, cDNA libraries were generated using either the Illumina TruSeq Stranded mRNA Sample Prep Kit protocol and dual-indexed, or the Universal Plus mRNA-seq NuQuant library prep protocol from Tecan, following the same protocol as Amar et al., 2024[5].

The experimental methods for the remaining 135 whole blood samples, seven of which were collected and processed at the Miami UDN site, are described in Arriaga et al., 2025[2]. In short, at Stanford, the cDNA libraries were generated using a Biomek i7 Liquid Handler robot with Tecan library-specific scripts, and pooled libraries were sequenced as 2x150bp paired-end reads on an Illumina Novaseq S2 Flow Cell. At the Miami UDN site, cDNA libraries were generated using the Illumina Stranded Total RNA Prep, and pooled libraries were sequenced on an Illumina NextSeq 550. As outlined in Arriaga et al., 2025[2], after sequencing, we removed 37 samples from further analysis due to missing information or insufficient RNA quality. Our resulting whole blood cohort consisted of 385 samples from 385 individuals.

Of all fibroblast samples, 134 of the total 139 samples originated from the Broad Center for Mendelian Genetics. cDNA libraries were generated using the Illumina TrueSeq Stranded mRNA Sample Prep Kit protocol and dual-indexed. The libraries were quantified after enrichment using Quant-iT PicoGreen (1:200 dilution). After normalizing samples to 5 ng/uL, the set was pooled and quantified using the KAPA Library Quantification Kit for Illumina Sequencing Platforms. Samples were then pooled and sequenced on Illumina Novaseq. For 108 samples, each run generated 101-bp paired-end reads, while for 30 samples, each run generated 151-bp paired-end reads. An eight-bp index barcode was generated for all samples.

The remaining five fibroblast samples, of which B1 was one, were prepared and sequenced by the UCLA Technology Center for Genomics and Bioinformatics using methods outlined in Lee et al., 2020[6]. Prior to sequencing, primary dermal fibroblasts were established from B1's skin biopsies. Cells were maintained at 37°C in a humidified atmosphere containing 5% CO_2 and cultured in standard growth medium composed of Dulbecco's Modified Eagle Medium supplemented with 10% fetal bovine serum and 1X Penicillin-Streptomycin-Glutamine. Adherent fibroblasts were then harvested from T75 flasks by enzymatic dissociation using TrypLE Express. Cells were washed with phosphate-buffered saline, pelleted by centrifugation, and prepared for RNA extraction. For RNA-sequencing, total RNA was isolated using the Maxwell RSC Instrument in conjunction with the Maxwell RSC simplyRNA Cells Kit, following the manufacturer's protocol. RNA concentration was measured using the Qubit Fluorometer, and RNA integrity was assessed with the Agilent Bioanalyzer. RNA-sequencing libraries were then sequenced using the Illumina NovaSeq 6000 platform, generating a minimum of 100 million paired-end reads (150 bp) per sample.

Pipeline

Transcriptome quality control and alignment

The computational pipelines for quality control and alignment 287 of the whole blood samples are outlined in Ungar et al., 2024[3], while the remaining 128 samples were preprocessed as described in Arriaga et al., 2025[2]. In short, we generated FASTQ files by demultiplexing BCL data using bcl2fastq (https://emea.support.illumina.com/sequencing/sequencing_software/bcl2fastq-conversion-software.html). The 287 samples analyzed in Ungar et al., 2024[3] were aligned to the hg38 human reference genome using STAR (2.8.4a)[7] and the GENCODEv35[8] primary genome annotations, while the remaining 128 samples were aligned using STAR (version=2.7.10a)[7] and GENCODEv39[8] primary genome annotations. Adapters for all 385 samples were removed, and reads were trimmed using cutadapt (version=2.4)[9] (https://github.com/marcelm/cutadapt) and optical duplicates removed using Picard (http://broadinstitute.github.io/picard).

FASTQ files for 134 of the fibroblast samples were generated from BCL data using IlluminaBasecallsToFastq (https://gatk.broadinstitute.org/hc/en-us/articles/9570268630683-IlluminaBasecallsToFastq-Picard) or BCL Convert (https://support.illumina.com/sequencing/sequencing_software/bcl-convert.html). The DRAGEN RNA pipeline from the Illumina DRAGEN Bio-IT Platform (https://support-docs.illumina.com/SW/dragen_v42/Content/SW/DRAGEN/TPipelineIntro_fDG.htm) was used with *--enable-duplicate-marking* set to true, allowing for duplicate read notation. PolyG and polyA tails were trimmed using *--soft-read-trimmers polyg, polya*. The minimum number of polyA bases required for trimming was set using *--trim-polya-min-trim* 20, and polyg soft trimming is enabled by default. Ribosomal RNA was removed using *--rrna-filter-enable=true*. The FASTQ files were then aligned to the hg38 human reference genome using STAR (version=2.7.10b)[7] in the two-pass mode and the GENCODEv39[8] primary genome annotation. For all 134 fibroblast samples, adapters were removed, and reads were trimmed using the Illumina DRAGEN Bio-IT Platform and optical duplicates removed using Picard (http://broadinstitute.github.io/picard).

RNA processing for the remaining 5 fibroblast samples is outlined in Lee et al., 2020[6]. Raw FASTQ files of the five fibroblast samples processed at UCLA were aligned to the GRCh37 human reference genome using STAR (version 2.5.2b)[7] with default parameters and GENCODEv19[8] primary genome annotation. Two-pass mapping was performed to maximize alignments across novel junctions. Depletion of ribosomal RNA was evaluated using BWA-mem[10] by calculating the portion of read pairs that aligned to references for the complete sequences of 5S, 12, 16S, 18S, and 28S ribosomal RNAs. Picard (http://broadinstitute.github.io/picard) performed duplicate marking, and additional quality metrics were generated via RNA-SeQC v1.1.8[11]. Single-nucleotide variant calls from genome sequencing were compared to the RNA-sequencing data to ensure correct sample identity.

Splicing outlier calling

Following guidelines from FRASER[12], we generated splicing outliers separately for the whole blood and fibroblast cohorts. The whole blood cohort was processed using FRASER (version=1.14.0)[12], while the fibroblast samples were processed using FRASER

(version=1.6.1)[12]. For both analyses, the default filterExpressionAndVariability settings were used; specifically, the minimum read count in at least one sample was 20, and the minimum ΔΨ was 0.0.. The pipeline and conda environments for this analysis can be found at https://github.com/maurermaggie/Transcriptome_Wide_Splicing_Analysis/tree/main/FRASER_snakemake.

Analysis of minor intron retention

Arriaga et al., 2025[2] employed a transcriptome-wide approach examining intron retention outliers in minor intron-containing genes (MIGs) to identify individuals with rare, biallelic variants in the minor spliceosome. Their method identified five individuals with an excess (Z-score > 2) of significant intron retention outliers in MIGs. Four individuals harbored rare, biallelic variants in *RNU4ATAC*, a minor spliceosome snRNA previously associated with disease[13-15]. One individual, known in this study as A1, was found to have rare biallelic variants in *RNU6ATAC*. As minor spliceopathies are characterized by specific retention of U12-type introns, we refined the method in Arriaga et al., 2025[2] to filter for minor intron retention (as opposed to intron retention *of any type* in MIGs).

Following Arriaga et al., 2025[2], junctions were defined as significant if their |ΔΨ| was greater than or equal to the default value suggested by FRASER[12] (0.3), and their adjusted p-value (q) after false discovery rate (FDR) correction was less than 0.05. ΔΨ is a normalization metric from FRASER[12] that is similar to a z-score. For a given intron from a specified sample, ΔΨ measures the difference between the observed and expected Ψ value.
The resulting significant ($q < 0.05$ and $|\Delta\psi| >= 0.3$) outliers were then filtered to only those of type θ, which captures partial or full intron retention. In order to filter for minor intron retention, we used the Homo_sapiens_intron download from the Minor Intron Database[16]. (https://midb.pnb.uconn.edu/return_downloads.php?species_var=Homo+sapiens). This database contains information on introns of the following types: major-like, minor-like, minor, non-canonical, major-hybrid, and minor-hybrid. We filtered to only introns with an intron_class of minor, then selected the following fields: gene_symbol, ensembl_gene_id, intron_start, and intron_end. We then filtered the significant θ introns for those whose gene name, Ensembl ID[17]

matched the first two aforementioned fields, and whose intron start and end matched the latter two fields ± 1. Similar to Arriaga et al., 2025[2], we defined individuals as having an excess of minor intron retention outliers if they contained a number of minor intron retention outliers greater than two standard deviations above the mean. The code used to filter our FRASER[12] outliers can be found at: https://github.com/maurermaggie/Transcriptome_Wide_Splicing_Analysis/tree/main/run_results_phenotype_paper.

References

1. Splinter K, Adams DR, Bacino CA, Bellen HJ, Bernstein JA, Cheatle-Jarvela AM, et al. Effect of Genetic Diagnosis on Patients with Previously Undiagnosed Disease. N Engl J Med. 2018 Nov 29;379(22):2131–9. Available from: https://doi:10.1056/NEJMoa1714458

2. Arriaga MT, Mendez R, Ungar RA, Bonner DE, Matalon DR, Lemire G, et al. Transcriptome-wide outlier approach identifies individuals with minor spliceopathies. Am J Hum Genet [Internet].2025 Oct 2;112(10):2458-2475. Available from: https://doi:10.1016/j.ajhg.2025.08.018

3. Ungar RA, Goddard PC, Jensen TD, Degalez F, Smith KS, Jin CA, et al. Impact of genome build on RNA-seq interpretation and diagnostics. Am J Hum Genet. 2024 July 11;111(7):1282–300. Available from: https://doi:10.1016/j.ajhg.2024.05.005

4. Cummings BB, Marshall JL, Tukiainen T, Lek M, Donkervoort S, Foley AR, et al. Improving genetic diagnosis in Mendelian disease with transcriptome sequencing. Sci Transl Med. 2017 Apr 19;9(386):eaal5209. Available from: https://doi:10.1126/scitranslmed.aal5209

5. Amar D, Nicole RG, Pierre MJ-B, Dam B, Surendra D, Courtney D, et al. Temporal dynamics of the multi-omic response to endurance exercise training. Nature. 2024 May;629(8010):174–183. Available from: https://doi:10.1038/s41586-023-06877-w

6. Lee H, Huang AY, Wang LK, Yoon AJ, Renteria G, Eskin A, et al. Diagnostic utility of transcriptome sequencing for rare Mendelian diseases. Genet Med Off J Am Coll Med Genet. 2020 Mar;22(3):490–9.

7. Dobin A, Davis CA, Schlesinger F, Drenkow J, Zaleski C, Jha S, et al. STAR: ultrafast universal RNA-seq aligner. Bioinforma Oxf Engl. 2013 Jan 1;29(1):15–21. Available from: https://doi:10.1093/bioinformatics/bts635

8. Frankish A, Diekhans M, Jungreis I, Lagarde J, Loveland JE, Mudge JM, et al. GENCODE 2021. Nucleic Acids Res. 2021 Jan 8;49(D1):D916–23. Available from: https://doi:10.1093/nar/gkaa1087

9. Martin M. Cutadapt removes adapter sequences from high-throughput sequencing reads. EMBnet.journal [Internet]. 2011 Oct 2;17, 10–12. Available from: https://doi:10.14806/ej.17.1.200

10. Li H, Durbin R. Fast and accurate short read alignment with Burrows-Wheeler transform. Bioinforma Oxf Engl. 2009 July 15;25(14):1754–60. Available from: https://doi.org/10.1093/bioinformatics/btp324

11. DeLuca DS, Levin JZ, Sivachenko A, Fennell T, Nazaire MD, Williams C, et al. RNA-SeQC: RNA-seq metrics for quality control and process optimization. Bioinforma Oxf Engl. 2012 June 1;28(11):1530–2. Available from: https://doi.org/10.1093/bioinformatics/bts196

12. Mertes C, Scheller IF, Yépez VA, Çelik MH, Liang Y, Kremer LS, et al. Detection of aberrant splicing events in RNA-seq data using FRASER. Nat Commun. 2021 Jan 22;12(1):529. Available from: https://doi.org/10.1038/s41467-020-20573-7

13. Merico D, Roifman M, Braunschweig U, Yuen RK, Alexandrova R, Bates A, et al. Compound heterozygous mutations in the noncoding RNU4ATAC cause Roifman Syndrome by disrupting minor intron splicing. Nat Commun. [Internet]. 2015 Nov 2;6, 8718. Available from: https://doi.org/10.1038/ncomms9718

14. Farach LS, Little ME, Duker AL, Logan CV, Jackson A, Hecht JT, et al. The expanding phenotype of RNU4ATAC pathogenic variants to Lowry Wood syndrome. Am J Med Genet [Internet]. 2018 Feb;.76, 465–469. Available from: https://doi.org/10.1002/ajmg.a.38581

15. Padgett RA, Shukla GC. A revised model for U4atac/U6atac snRNA base pairing. RNA [Internet]. 2002 Feb;125–128. Available from: https://doi.org/10.1017/s1355838202017156

16. Olthof AM, Hyatt KC, Kanadia RN. Minor intron splicing revisited: identification of new minor intron-containing genes and tissue-dependent retention and alternative splicing of minor introns. BMC Genomics [Internet]. 2019 Aug 30;20(1):686. Available from: https://doi.org/10.1186/s12864-019-6046-x

17. Dyer SC, Austine-Orimoloye O, Azov AG, Barba M, Barnes I, Barrera-Enriquez VP, et al. Ensembl 2025. Nucleic Acids Res [Internet]. 2025 Dec 4;53(D1), D948–D957. Available from: https://doi.org/10.1093/nar/gkae1071
