# Supplementary material for: Bi-allelic RNU6ATAC variants cause a minor spliceopathy characterized by transcriptome-wide minor intron retention and multisystem manifestations
Source: HGG Adv. 2026 Mar 9;7(2):100588. doi: 10.1016/j.xhgg.2026.100588 (PMC13049632; doi:10.1016/j.xhgg.2026.100588)
Supplement: Document S2. Article plus supplemental information [file mmc2.pdf]

HGG Advances

BRIEF COMMUNICATION

Bi-allelic *RNU6ATAC* variants cause a minor spliceopathy characterized by transcriptome-wide minor intron retention and multisystem manifestations

Rodrigo Mendez,[1,18,20,*] Taylor M. Arriaga,[2,18] Jialan Ma,[3] Devon E. Bonner,[4] Sara Emami,[2] Rebecca J. Levy,[5] Afaf Alsagheir,[6,7] Bader Alhaddad,[8] Khadijah Bakur,[8] Rachel A. Ungar,[2,9] Dena R. Matalon,[4] Alexander M. Miller,[10] Jonathan Nguyen,[10] Kevin S. Smith,[10] Stuart A. Scott,[10,11] Linda Liao,[11] Zena Ng,[11] Shruti Marwaha,[1] Alistair Ward,[12,13] Undiagnosed Diseases Network, Genomics Research to Elucidate the Genetics of Rare Diseases Consortium, Danica Novacic,[14] Fowzan S. Alkuraya,[7,8,15] Jonathan A. Bernstein,[4] Vijay S. Ganesh,[3,16,19] Anne O'Donnell-Luria,[3,17,19] Stephen B. Montgomery,[2,10,19] and Matthew T. Wheeler[1,19,*]

Summary

We report three individuals with bi-allelic variants in RNU6ATAC, which encodes the U6atac minor spliceosomal small nuclear RNA (snRNA), causing a multisystem minor spliceopathy. Through RNA sequencing analysis, we identified a distinctive excess of minor intron retention (MIR) in two unrelated individuals, which guided the identification of bi-allelic *RNU6ATAC* variants. The discovery cohort presented with variable multisystem manifestations. One individual presented with refractory epilepsy, microcephaly, developmental delay, ataxia, bilateral toe syndactyly, hypereosinophilia, and short stature, whereas the other exhibited failure to thrive, short stature, primary hypothyroidism, combined variable immunodeficiency, eosinophilic colitis, ichthyosis vulgaris, scoliosis, and chronic inflammatory demyelinating polyneuropathy without neurodevelopmental involvement. Despite organ-specific variation, both individuals displayed impaired growth and eosinophil-driven inflammation. Recently, we identified a third affected individual from an independent cohort whose phenotype bridges these features, combining microcephaly, growth failure with severe immunodeficiency, and skeletal abnormalities. The distinctive excess of MIR outliers in the discovery cohort supports minor spliceosome dysfunction, mirroring the molecular signature of RNU4ATAC-opathy. These findings nominate *RNU6ATAC* as a disease-associated gene, defining an expanded clinical spectrum of minor spliceopathies. Our study supports the power of integrating genomic and transcriptomic approaches for diagnosing splicing disorders and highlights the critical role of spliceosomal snRNAs in human disease.

Introduction

Humans have two spliceosome machineries, characterized by unique consensus sequences and ribonucleoprotein (RNP) complexes.[1] The major spliceosome removes over 99.5% of introns, and the minor spliceosome processes the remaining <0.5% (~770 introns in 715 essential genes).[1] The minor spliceosome comprises five small nuclear RNAs (snRNAs): U11, U12, U4atac, U6atac, and U5, the latter of which is shared with the major spliceosome. U11 and U12 form a stable U11/U12 di-snRNP that recognizes the minor 5′ splice site and branchpoint sequence early in spliceosome assembly.[2] U4atac and U6atac create a bimolecule through extensive base pairing in the conserved stem I and II regions of U4atac.[3,4] Disruption of U4atac destabilizes this complex, preventing proper trimolecule assembly with U5, and thereby compromising minor intron recognition and processing.[5] Once activated, U6atac dissociates from U4atac and establishes new base-pairing interactions with U12 and the branchpoint of the intron, forming the catalytic core of the spliceosome.[6] Overall, the integrity of each snRNA is essential for minor intron splicing, and disruption in any of these components could result in minor intron retention (MIR).[7]

Pathogenic variants in minor spliceosomal snRNAs have been identified in *RNU4ATAC*[8] (RNA, U4atac small

[1]Division of Cardiovascular Medicine, Department of Medicine, Stanford University, Stanford, CA, USA; [2]Department of Genetics, Stanford University, Stanford, CA, USA; [3]Program in Medical and Population Genetics, Broad Institute of MIT and Harvard, Cambridge, MA, USA; [4]Division of Medical Genetics, Department of Pediatrics, Stanford University School of Medicine, Stanford, CA, USA; [5]Division of Child Neurology, Department of Neurology and Neurological Sciences, Stanford University, Stanford, CA, USA; [6]Department of Pediatrics, King Faisal Specialist Hospital and Research Centre, Riyadh, Saudi Arabia; [7]College of Medicine, Alfaisal University, Riyadh, Saudi Arabia; [8]Lifera Omics, Riyadh, Saudi Arabia; [9]Stanford Center for Biomedical Ethics, Stanford University, Stanford, CA, USA; [10]Department of Pathology, Stanford University, Stanford, CA, USA; [11]Clinical Genomics Laboratory, Stanford Medicine, Stanford, CA, USA; [12]Department of Human Genetics, University of Utah, Salt Lake City, UT, USA; [13]Frameshift Labs, Cambridge, MA, USA; [14]Undiagnosed Diseases Program, National Human Genome Research Institute, National Institutes of Health, Bethesda, MD, USA; [15]Department of Translational Genomics, Genomic Medicine Center of Excellence, King Faisal Specialist Hospital and Research Center, Riyadh, Saudi Arabia; [16]Department of Neurology, Brigham and Women's Hospital, Boston, MA, USA; [17]Division of Genetics and Genomics, Boston Children's Hospital, Boston, MA, USA
[18]These authors contributed equally
[19]Senior author
[20]Lead contact
*Correspondence: mendezh@stanford.edu (R.M.), wheelerm@stanford.edu (M.T.W.)
https://doi.org/10.1016/j.xhgg.2026.100588.
© 2026 The Authors. Published by Elsevier Inc. on behalf of American Society of Human Genetics.
This is an open access article under the CC BY license (http://creativecommons.org/licenses/by/4.0/).

nuclear [MIM: 601428]) and *RNU12*[9] (RNA, U12 small nuclear [MIM: 620204]). Bi-allelic variants in *RNU4ATAC* were reported in microcephalic osteodysplastic primordial dwarfism type 1 (MOPD1 [MIM: 210710]), Roifman (RFMN [MIM: 616651]), and Lowry-Wood (LWS [MIM: 226960]) syndromes,[10] which are now considered RNU4atac-opathies. These disorders encompass a clinical spectrum, including severe short stature or spondyloepiphyseal dysplasia, microcephaly, neurodevelopmental delay, retinal dystrophy, and immunodeficiency.[11] Bi-allelic variants in *RNU12* cause autosomal recessive spinocerebellar ataxia-33[9] (SCAR33 [MIM: 620208]), and CDAGS syndrome ([MIM: 603116]) (an acronym for craniosynostosis, delayed fontanel closure, anal anomalies, genitourinary, and skin abnormalities).[12] These disorders illustrate how defects in the minor spliceosome lead to a broad array of clinical phenotypes, from multisystem skeletal dysplasia with brain and immune involvement to more organ-restricted effects, as seen in SCAR33.

In this study, we identified bi-allelic *RNU6ATAC* (RNA, U6atac small nuclear [MIM: 601429]) variants in three unrelated individuals, guided by MIR outliers in the discovery cohort. We aim to demonstrate that *RNU6ATAC* variants cause transcriptome-wide minor splicing disruption and correlate this dysfunction with the observed phenotypes.

Material and methods

Ethics declaration

Undiagnosed Diseases Network (UDN) participants were enrolled under the Stanford University institutional review board (IRB) (protocols 47026 and 60837) and the National Human Genome Research Institute IRB (protocol 15-HG-0130). All individuals provided written informed consent. The replication cohort was enrolled under King Faisal Specialist Hospital and Research Center (KFSHRC) IRB approval (2230016), with consent for publication of identifiable information obtained under protocol Research Advisory Council (RAC) number 2080006.

Transcriptome analysis

RNA sequencing (RNA-seq) methods for 385 whole-blood[5,13] and 139 fibroblast samples are detailed in supplemental material 1. We adapted the outlier detection framework described in Arriaga et al.[5] to specifically target MIR outliers. This refinement was motivated by the distinct architecture of minor intron-containing genes (MIGs), which typically contain multiple introns,[1] only a small fraction of which are minor (U12-type) introns.[1] Given that minor spliceopathies are characterized by the retention of minor introns,[14] we refined our analysis to filter out canonical intron retention events within MIGs.

We applied FRASER[15] separately to the whole-blood and fibroblast cohorts. Outliers were filtered to retain only significant θ outliers (representing partial or full intron retention) that met the significance thresholds established in Arriaga et al.[5] We then quantified significant θ outliers in introns classified as "minor" in the Minor Intron Database,[1] thereby calculating the number of MIR outliers per individual. Individuals were defined as having an excess of MIR if their count of MIR outliers was greater than 2 standard deviations from the mean of their respective tissue type.

The code used to filter, create, and analyze FRASER[15] outliers is available in the FRASER_snakemake and run_results_phenotype_paper (https://github.com/maurermaggie/Transcriptome_Wide_Splicing_Analysis).

Genome sequencing and reanalysis

Whole-genome sequencing (WGS) was performed for individuals A1 and B1 and their respective family members by Baylor Genetics through the UDN. Individual C1 and his parents underwent clinical WGS at Centogene, as requested by KFSHRC. Additional details can be found in supplemental material 1. WGS was considered essential for this study because *RNU6ATAC* is not targeted by major commercial whole-exome sequencing (WES) kits (according to the University of California, Santa Cruz [UCSC][16] "Exome Capture Probesets" track; GRCh38, chr9:134,164,439–134,164,564), and this was confirmed by the absence of sequencing coverage across the aggregated exome dataset in gnomAD.[17,18] For the discovery cohort, genomic reanalysis was conducted using the Mosaic genomic platform (https://frameshift.io/mosaic) on the latest UDN dataset, which included WGS data from 5,323 individuals. Variant prioritization focused on rare variants (allele frequency [AF] <1% in gnomAD version 4.1.0[17,18]) located in minor spliceosome snRNAs.

Results

Transcriptome analysis

A recent study by Arriaga et al.[5] employed FRASER[15] to examine transcriptome-wide outlier patterns in 385 whole-blood samples from the UDN and Genomics Research to Elucidate the Genetics of Rare diseases (GREGoR) consortium. This approach identified five individuals with an excess of intron retention events in MIGs (mean: 286.6, median: 270, interquartile range [IQR]: 252–313) compared with the rest of the cohort (mean: 1.7, median: 0, IQR: 0–2), including six close relatives of the five aforementioned individuals. Genomic analysis of the five individuals with an excess of intron retention outliers in MIGs revealed that four had RNU4atac-opathies. The fifth individual (A1), who remained undiagnosed, had 252 intron retention events in MIGs.

To better reflect the molecular etiology of minor spliceopathies, we refined this method to quantify retention specifically in minor (U12-type) introns, rather than all introns within MIGs. When applied to the original 385-sample whole-blood cohort, our refined method recapitulated the same five outliers. These individuals exhibited a profound excess of MIR (mean: 288.8, median: 268 events, IQR: 254–309) compared with the remainder of the cohort (mean: 0.1, median: 0, IQR: 0–0; Figure 1A). Specifically, individual A1 showed a distinct outlier profile (*Z* score: 7.7), harboring 254 MIR outliers across 142 MIGs (Figure 1A). Importantly, focusing specifically on minor introns significantly reduced background noise, and the rate of retention in unaffected samples dropped from a mean of 1.7 events in the original analysis to 0.1 events in our refined analysis.

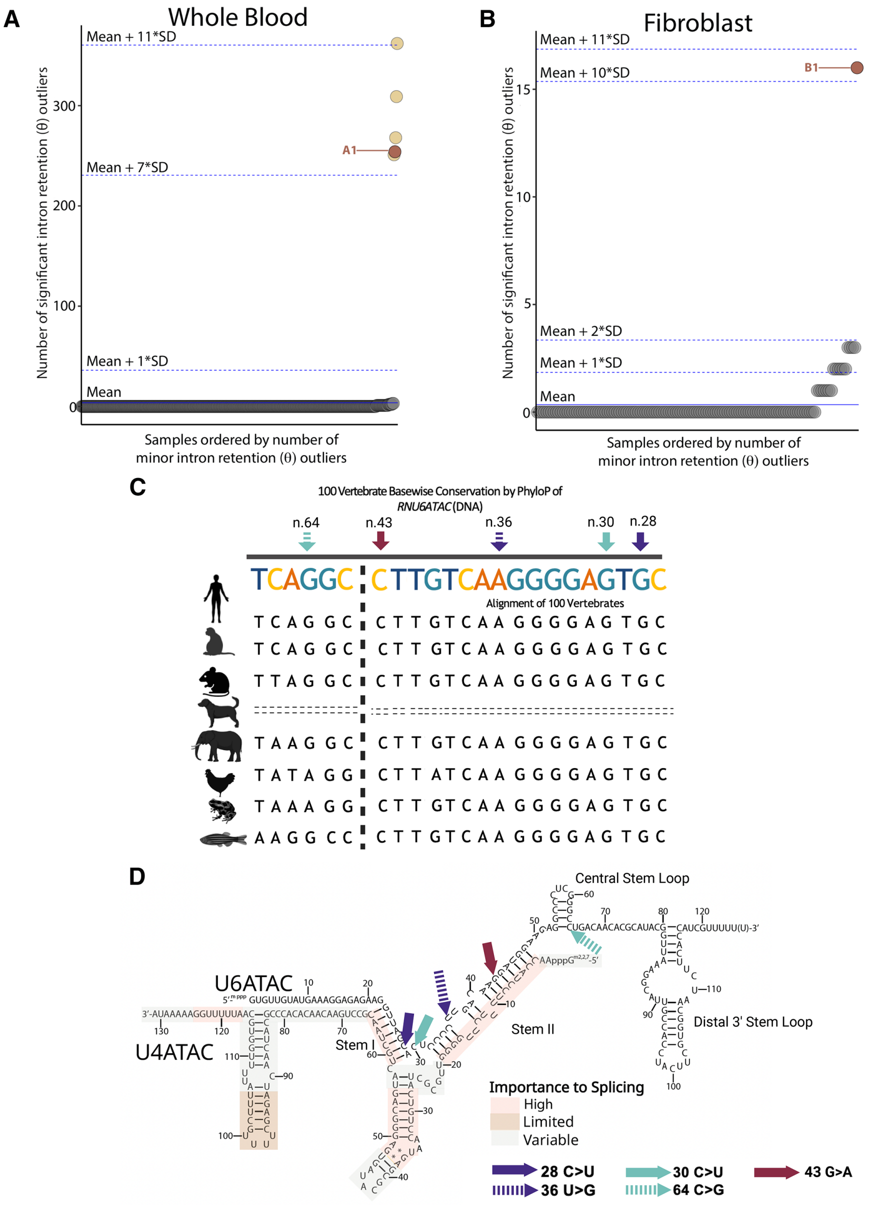

Figure 1. Rare, conserved bi-allelic *RNU6ATAC* variants are associated with an excess of significant minor intron retention (*θ*) outliers

(A) Outlier individual A1 with an excess of minor intron retention (*θ*) outliers. Plot showing the number of significant ($q < 0.05$, abs($\Delta\Psi$) ≥ 0.3) minor intron retention (*θ*) outliers in our 385-person whole-blood cohort. Each dot represents an individual, and the *y* axis position represents the number of significant minor intron retention (*θ*) outliers detected in that individual. The *x* axis is ordered by the number of significant minor intron retention (*θ*) outliers per individual. The red, labeled circle represents individual A1 with rare, bi-allelic variants in *RNU6ATAC*, while the yellow circles represent individuals with RNU4atac-opathy.

(B) Outlier individual B1 with an excess of minor intron retention (*θ*) outliers. Plot showing the number of significant ($q < 0.05$, abs($\Delta\Psi$) ≥ 0.3) minor intron retention (*θ*) outliers in our 139-person fibroblast cohort. Each dot represents an individual, and the *y* axis position represents the number of significant minor intron retention (*θ*) outliers detected in that individual. The *x* axis is ordered by the number of significant minor intron retention (*θ*) outliers per individual. The red, labeled circle represents individual B1 with rare, bi-allelic variants in *RNU6ATAC*.

(C) Conservation of the *RNU6ATAC* genomic region across vertebrates. Nucleotide-level conservation scores for the *RNU6ATAC* gene were computed using PhyloP across 100 vertebrate species. Representative aligned sequences from human, rhesus macaque, mouse, dog, elephant, chicken, *Xenopus tropicalis*, and zebrafish are shown. The two variants identified in individual A1 are marked by purple solid and dashed arrows, highlighting their position within highly conserved regions. The two variants identified in individual B1 are marked by teal solid and dashed arrows, highlighting their position within highly conserved regions. The variant found in individual C1 is indicated by a dark red arrow, showing its location within highly conserved regions.

(D) Disruption of U4atac-U6atac RNA duplex involved in minor spliceosome catalysis. A secondary structure model of the RNA duplex formed between U4atac and U6atac is necessary for creating the catalytic core of the minor spliceosome. The two variants identified in individual A1 are marked with purple solid and dashed arrows. The two variants identified in individual B1 are marked with teal solid and dashed arrows. The variant found in individual C1 is indicated by a dark red arrow. Nucleotide positions in U4atac/U6atac bimolecule are colored according to their functional importance in minor splicing, as the legend indicates.

We subsequently extended this framework to a fibroblast cohort of 139 individuals from the Stanford UDN clinical site and the GREGoR consortium. This analysis identified a single additional outlier, individual B1, for whom fibroblasts were the only available tissue sample. This individual exhibited an excess of MIR outliers (*Z* score: 10.4). Individual B1 had 16 MIR events in 14 MIGs, whereas the rest of the cohort had, on average, less than one MIR event (mean: 0.2, median: 0, IQR: 0–0) in less than one MIG (mean: 0.2, median: 0, IQR: 0–0) (Figure 1B).

Genome reanalysis

We identified bi-allelic *RNU6ATAC* (GenBank: NR_023344.1) variants in all three affected individuals, consistent with autosomal recessive inheritance (Table 1). In the discovery cohort, individual A1 was compound heterozygous for variants affecting the U4atac/U6atac stem I region[5] (NR_023344.1: n.28C>T, maternal inheritance) and the U4atac/U6atac stem II region[5] (NR_023344.1:n.36T>G, paternal inheritance). Individual B1 harbored bi-allelic variants disrupting the U4atac/U6atac stem I/II boundary (NR_023344.1: n.30C>T,

Table 1. Summary of genetic findings for both affected individuals

Feature	Individual A1		Individual B1		Individual C1	
	Compound heterozygous		Compound heterozygous		Homozygous	
Genomic coordinate (hg38)	9-134164537-G-A	9-134164529-A-C	9-134164535-G-A	9-134164501-G-C	9-134164522-C-T	9-134164522-C-T
HGVS.C (NR_023344.1)	n.28C>T	n.36T>G	n.30C>T	n.64C>G	n.43G>A	n.43G>A
Inheritance	maternal	paternal	maternal	paternal	maternal	paternal
gnomAD version 4.1.0 allele frequency	0.0000789	absent	0.00000658	absent	0.00001313	0.00001313
gnomAD version 4.1.0 homozygotes	0	NA	0	NA	0	0
CADD score	21	18	21	19	19	19
PhyloP100 score	9.55	7.12	7.62	3.98	4.72	4.72
ClinVar classification	VUS	VUS	VUS	VUS	VUS	VUS
ClinVar accession number	SCV007299091	SCV007299092	SCV007299093	SCV007299094	SCV007334818	SCV007334818
snRNA region affected	U6atac/U4atac stem I pairing region	U6atac/U4atac stem II pairing region	stem I/II boundary of the U4atac/U6atac bimolecule	central stem-loop region	U6atac/U4atac stem II pairing region	U6atac/U4atac stem II pairing region
RNA sequencing sample type	whole blood		fibroblasts		NA	
Transcriptomic θ outliers	254 minor intron retention outliers		16 minor intron retention outliers		NA	

All identified variants are rare and affect evolutionarily conserved positions. Computational predictions support their potential deleterious effects. The table includes ClinVar classifications, ClinVar accession numbers, and transcriptomic outlier status. CADD, combined annotation-dependent depletion; NA, not applicable; snRNA, small nuclear RNA; VUS, variant of uncertain significance.

maternal inheritance), which was previously reported in ClinVar[19] as of uncertain significance (ClinVar: RCV004764396.1), and the Central Stem-loop (NR_023344.1: n.64C>G, paternal inheritance), a critical binding site for the splicing factor CENATAC[20] (centrosomal AT-AC splicing factor [MIM: 6200142]).

In the replication cohort, individual C1 was homozygous for a U4atac/U6atac stem II region variant (NR_023344.1: n.43G>A),[10] with both parents genotyped as carriers. Structurally, n.43 base pairs with *RNU4ATAC* position n.8, a recognized mutational hotspot,[10] indicating that disrupting this specific interaction destabilizes the minor spliceosome complex. Detailed genetic findings are included in supplemental material 2.

All identified variants are absent or extremely rare in gnomAD version 4.1.0,[17,18] and according to PhyloP100,[16] they impact highly conserved nucleotides predicted to be essential for minor spliceosome assembly and function (Figures 1C and 1D).

Notably, all *RNU6ATAC* variants identified in this study yielded combined annotation-dependent depletion (CADD[21]) Phred scores between 18 and 21, well above the region-specific threshold of 11.44 set by Tenywa et al. for pathogenic non-coding RNAs (ncRNAs).[22]

To identify additional affected individuals, we submitted the *RNU6ATAC* gene to GeneMatcher,[23] which yielded one non-informative research-interest match, and engaged international collaborators, leading to the inclusion of individual C1.

Clinical overview of the study cohort

The discovery cohort comprises two individuals with distinct multisystem presentations. Individual A1 is a 14-year-old female with intrauterine growth restriction, microcephaly, refractory epilepsy, and cerebral structural anomalies. Her course is further complicated by ataxia, autism, severe intellectual disability, and marked peripheral eosinophilia (Figures 2A and 2B). Individual B1 is a 30-year-old male with a multisystem disorder characterized by prominent immune dysfunction, endocrinopathy, and ectodermal abnormalities. His clinical history includes primary hypothyroidism, failure to thrive, bronchiectasis, alopecia universalis, chronic inflammatory demyelinating polyneuropathy, and combined variable immunodeficiency (CVID), without neurodevelopmental involvement (Figures 3A and 3B). The replication cohort includes individual C1, a 17-year-old male who presents with microcephaly, growth failure, developmental delay, immunodeficiency, and severe skeletal dysplasia (Figures 4A and 4B). Comprehensive clinical descriptions are provided in supplemental material 2.

Discussion

We report three unrelated individuals with bi-allelic variants in *RNU6ATAC*, the gene encoding the U6atac snRNA of the minor spliceosome.[1] These cases expand the spectrum of phenotypes associated with defects in the minor spliceosome. Notably, the clinical features observed across these three individuals span many organ systems and resemble the pleiotropic presentation of known minor spliceopathies.[8,9,24] Typical findings in RNU4atac-opathies (MOPD1, Roifman, and Lowry-Wood syndromes)[8,10,25] include severe growth restriction, microcephaly, skeletal dysplasia, and cognitive impairment, with variable additional involvement of the brain, immune system, cardiovascular system, eyes, skin, gastrointestinal tract, hearing, and endocrine organs,[8,10,25] while individuals with *RNU12* variants present with SCAR33[9] or CDAGS syndrome.[12]

The phenotype of individual A1 aligns with a severe, neurologically focused spectrum; she has microcephaly, profound developmental impairment with intractable epilepsy, and structural brain anomalies. Her short stature also aligns with the growth characteristic of RNU4atac-opathies.[8,10,25] The presence of ataxia mirrors the presentation of SCAR33.[9] Immune dysfunction is not prominent in A1; she did not have recurrent infections, but her hypereosinophilia suggests immune dysregulation.

In contrast, the phenotype of individual B1 lacked microcephaly, epilepsy, and cognitive impairment, but was defined by a predominantly systemic presentation featuring a profound CVID-like immunodeficiency (supplemental material 3). Beyond the shared clinical features of growth failure and eosinophil-related inflammation, the immunophenotype of individual B1 mirrors characteristics seen in RNU4atac-opathy,[26] especially severe B cell lymphopenia and hypogammaglobulinemia consistent with the B cell developmental block described by Heremans et al.[26] However, the immune profile of individual B1 is distinguished by significant T cell pathology not typically seen in Roifman syndrome,[26] including $CD4^+$ lymphopenia. Along with his autoimmune polyendocrinopathy-candidiasis-ectodermal dystrophy-like ectodermal features, we hypothesize that *RNU6ATAC* dysfunction might perturb MIGs regulating lymphocyte development more broadly, likely impairing both bone marrow B cell formation[26] and thymic T cell maturation checkpoints.

Importantly, identifying individual C1 fills a crucial gap in this phenotypic spectrum. Individual C1 exhibits a "bridging phenotype," which combines the constitutional growth failure and developmental delay of A1 with the severe immunodeficiency (pan-hypogammaglobulinemia, bronchiectasis) and autoimmunity (type 1 diabetes) observed in individual B1. This unification suggests that the neurodevelopmental and immune-skeletal presentations are not separate entities but rather endpoints of a continuous RNU6ATAC-opathy spectrum. We also emphasize the importance of ongoing identification of affected individuals and thorough molecular characterization to fully understand the genotype-phenotype correlations within this locus.

The *RNU6ATAC* variants identified in our cohort yielded CADD[21] Phred scores of 18–21, well above the

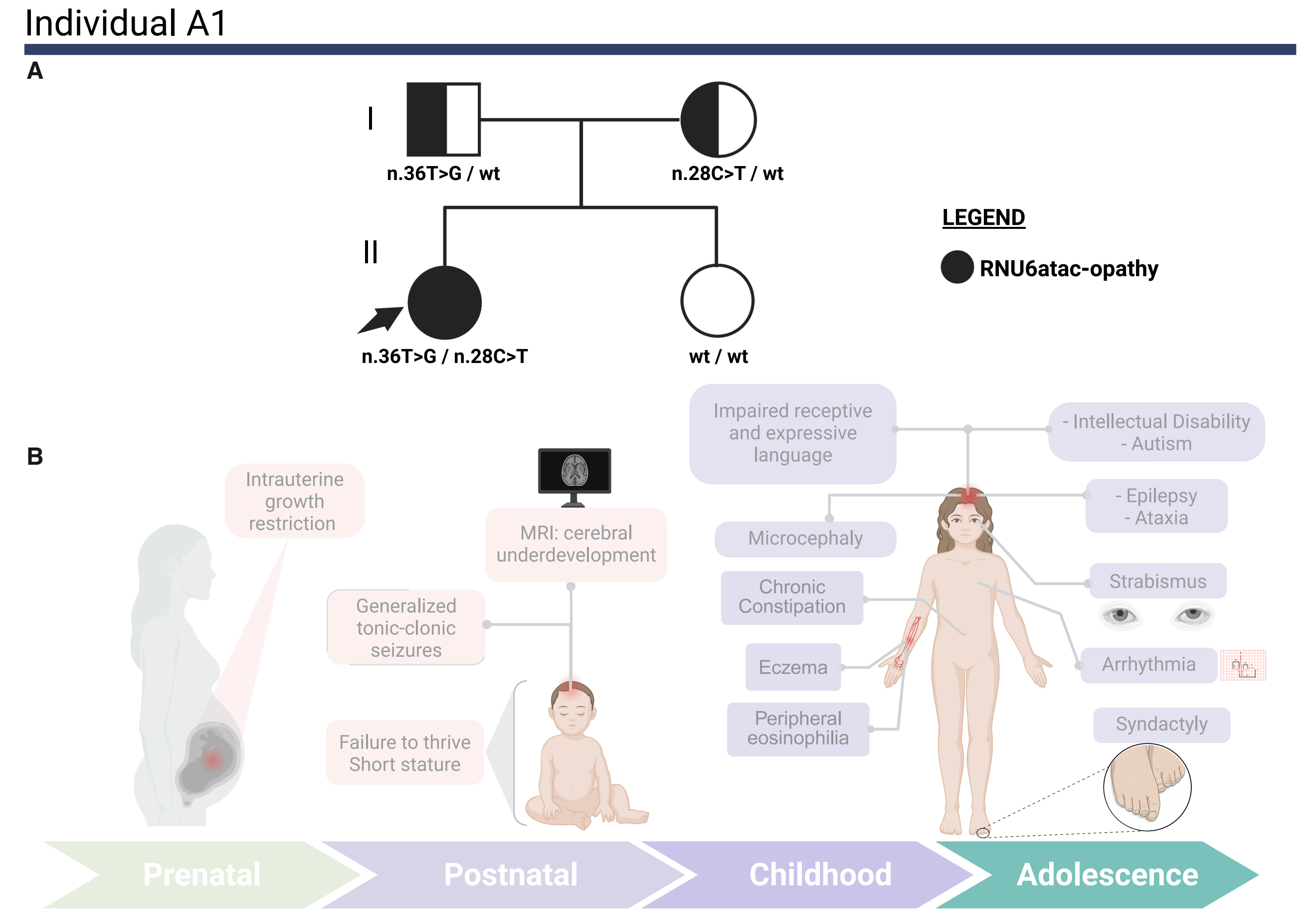

Figure 2. Pedigree and clinical features of individual A1, with compound heterozygous *RNU6ATAC* variants
(A) Pedigree of individual A1's family: the pedigree illustrates the segregation pattern of *RNU6ATAC* variants (NR_023344.1: n.36T>G and NR_023344.1: n.28C>T) in a compound heterozygous state in the proband (individual A1), who is marked with an arrow. The proband is represented by a fully black circle, indicating that she is affected by RNU6atac-opathy. The father is shown as a square half-filled in black, indicating carrier status for the NR_023344.1: n.36T>G variant. The mother is depicted as a circle half-filled in black, showing carrier status for the NR_023344.1: n.28C>T variant. The sister of the proband is represented by a white circle, consistent with a wild-type genotype for both variants, confirming she does not carry either variant. Segregation was confirmed through orthogonal Sanger sequencing.
(B) Illustration of key clinical features: a schematic representation of individual A1's major clinical manifestations across different life stages, from prenatal to her current age of 14 years.

11.44 threshold proposed by Tenywa et al. for prioritizing deleterious variants in ncRNA regions.[22] This range is consistent with the recurrent pathogenic *RNU4-2* insertion (CADD 20.8), supporting the notion that region-aware *in silico* scores can effectively flag deleterious changes in spliceosomal snRNAs.[22] Additionally, all five *RNU6ATAC* variants identified in the cohort map to conserved regions of the U6atac snRNA secondary structure, providing clues to how they may disrupt the minor spliceosome. The bi-allelic variants in A1 (NR_023344.1: n.28C>T and NR_023344.1: n.36T>G) likely impair the formation of the U4atac/U6atac di-snRNP complex by disrupting key base-pairing interactions in stem I and stem II.[3,20] Similarly, individual B1 harbored two variants (NR_023344.1: n.30C>T and NR_023344.1: n.64C>G) outside the canonical stem I and stem II domains. These positions may subtly destabilize RNA structure or disrupt interactions with accessory proteins such as CENATAC,[20] likely leading to qualitatively different effects on minor spliceosome function. Notably, individual C1 is homozygous for a variant in the U4atac/U6atac stem II region (NR_023344.1: n.43G>A) that base pairs directly with nucleotide n.8 of *RNU4ATAC*.[10] Since *RNU4ATAC* n.8 is a known pathogenic hotspot reported in multiple affected individuals with RNU4ATAC-opathy,[10] this provides structural evidence that disrupting this specific intermolecular interaction could interfere with the normal function of the minor spliceosome.

Transcriptome-wide analysis supports the hypothesis that the splicing defect in individuals A1 and B1 is specific to MIR, paralleling the selective splicing anomalies reported in RNU4atac-opathy.[5] This conserved transcriptomic pattern indicates a common mechanism of minor spliceopathies, providing molecular evidence that RNU6atac-opathy belongs in the expanding group of minor spliceopathies. In our study, the markedly

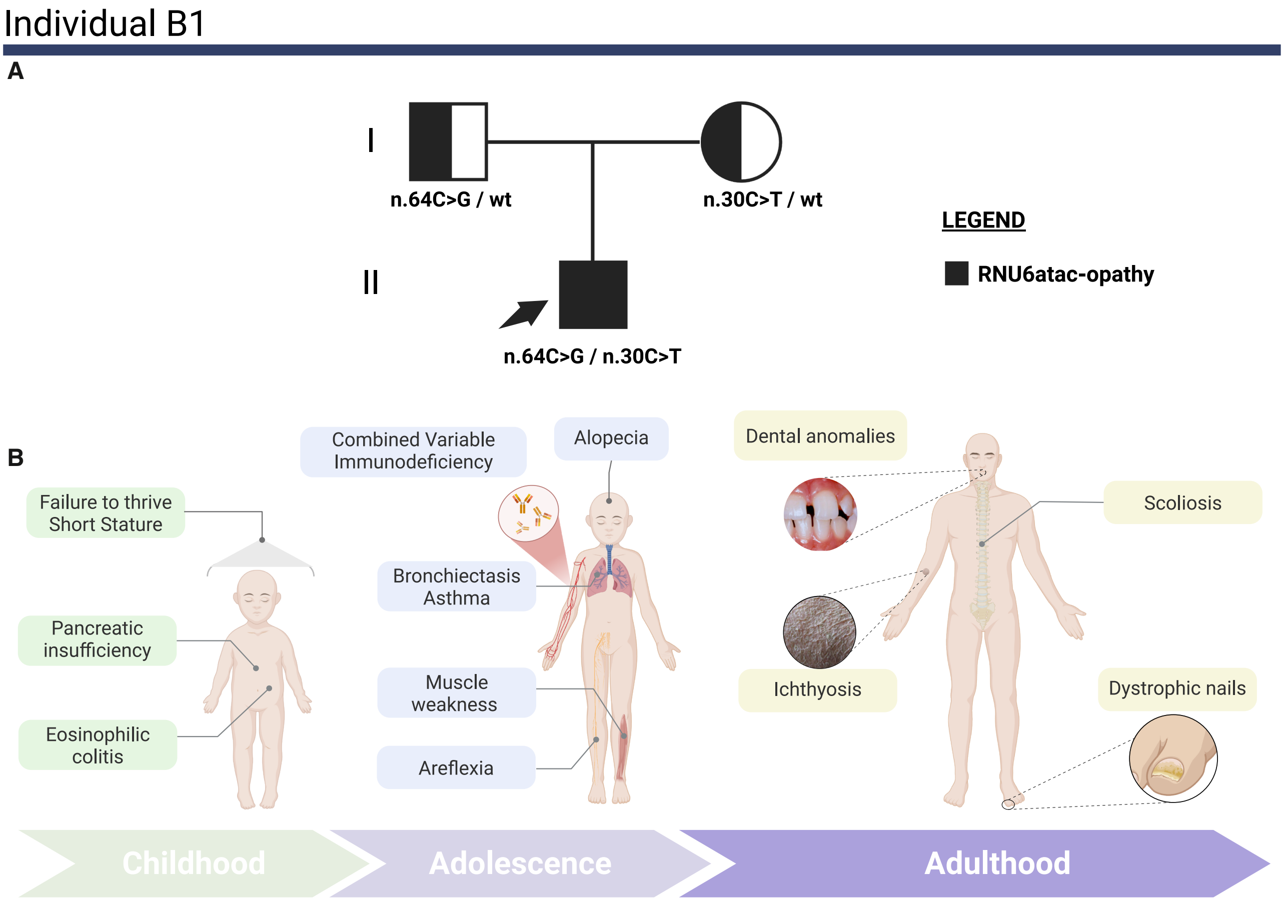

Figure 3. Pedigree and clinical features of individual B1 with compound heterozygous *RNU6ATAC* variants
(A) Pedigree of individual B1's family: the pedigree illustrates the inheritance pattern of *RNU6ATAC* variants (NR_023344.1: n.30C>T and NR_023344.1: n.64C>G) in a compound heterozygous state in the proband (individual B1), who is indicated by an arrow. The proband is represented by a fully black circle, indicating that he is affected by RNU6atac-opathy. The father is shown as a square half-filled in black, indicating carrier status for the NR_023344.1: n.64C>G variant. The mother is depicted as a circle half-filled in black, indicating carrier status for the NR_023344.1: n.30C>T variant. Segregation was confirmed through orthogonal Sanger sequencing.
(B) Illustration of key clinical features: a schematic representation of individual B1's major clinical manifestations across different life stages, from childhood through adolescence to adulthood.

higher excess of MIR outliers observed in whole blood (individual A1, 252 events) compared with fibroblasts (individual B1, 16 events) may reflect a convergence of tissue-specific transcript abundance and differential biological sensitivity, but we cannot discount intrinsic differences in the degree of impact of the variants.[14,27] Previous evidence shows that the transcriptional landscape varies by tissue; many MIGs are expressed at low levels in fibroblasts, which limits the pool of detectable transcripts for intron retention analysis relative to blood.[27] We acknowledge that a direct comparison would utilize paired samples; however, material was limited to blood for A1 and fibroblasts for B1. Our findings parallel those in RNU4atac-opathy,[5,27] in which hematopoietic lineages exhibit more severe U12-type intron mis-splicing than mesenchymal cells.[27] This aligns with the concept that the low-abundance minor spliceosome might create a bottleneck in rapidly dividing tissues.[14] Collectively, these data likely support a model in which *RNU6ATAC* variants reduce the functional reserve of the minor spliceosome below the threshold required for hematopoietic homeostasis, while remaining partially sufficient for basal splicing in fibroblasts.[27] More affected individuals will need to be identified to better understand genotype-phenotype correlations in this condition.

Finally, our findings underscore the necessity of WGS to detect variants in RNU6ATAC, which are missed by standard WES. Furthermore, we reveal a significant gap in rare disease transcriptomics. Since minor introns account for less than 0.5% of all introns in the human genome,[1] standard global retention metrics often overlook specific minor spliceosome dysfunctions. To capture this signal, we recommend that clinical bioinformatics pipelines incorporate targeted filtering that intersects splicing outliers with minor intron annotations.[1] We advocate adopting the FRASER-based[15] outlier detection framework used in Arriaga et al.[5] and refined in this study, as it effectively detects MIR outliers that would

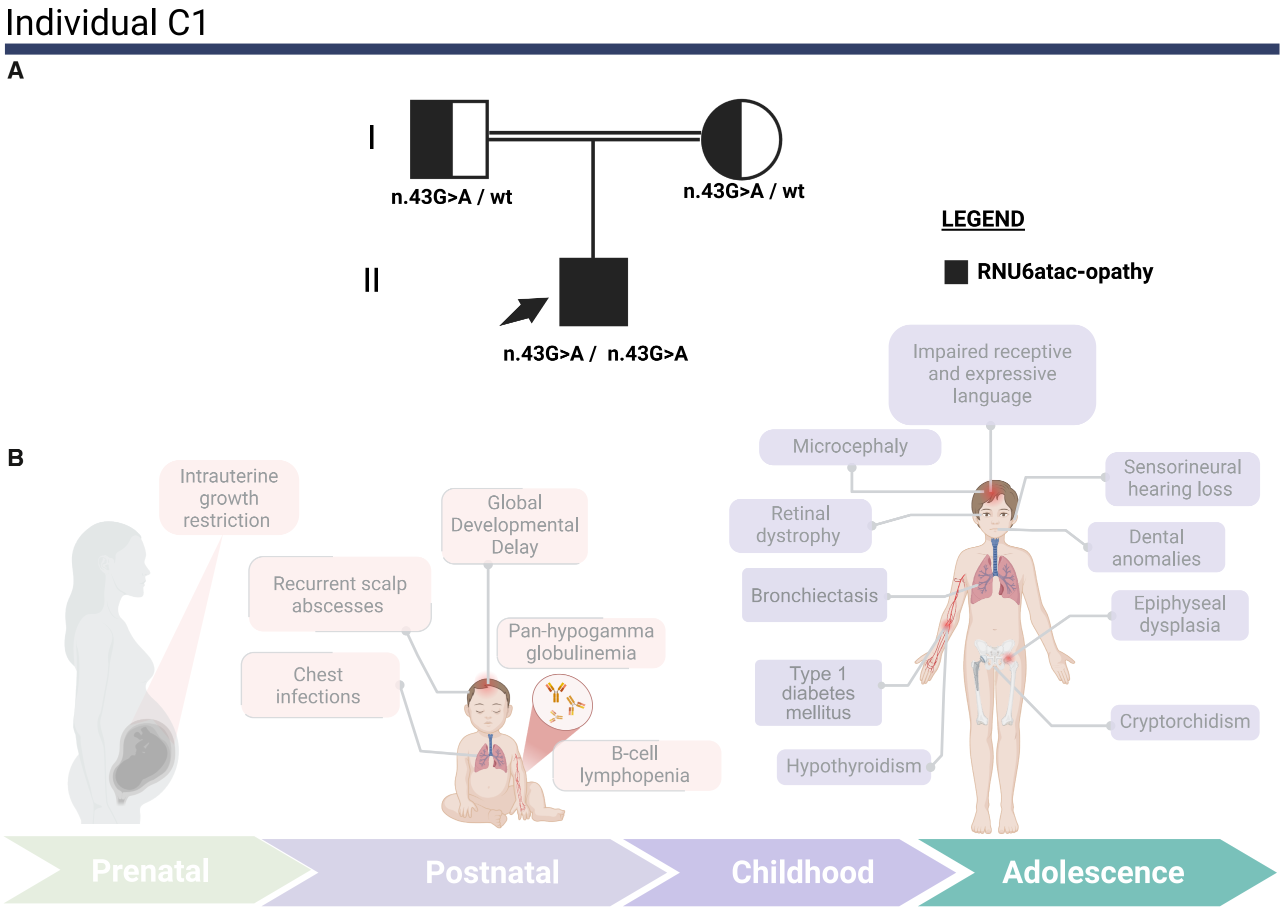

Figure 4. Pedigree and clinical features of individual C1 with a homozygous *RNU6ATAC* variant
(A) Pedigree of individual C1's family: the pedigree illustrates the inheritance pattern of *RNU6ATAC* variants (NR_023344.1: n.43G>A) in a homozygous state in the proband (individual C1), who is indicated by an arrow. The proband is represented by a fully black circle, indicating that he is affected by RNU6atac-opathy. The father is shown as a square half-filled in black, indicating carrier status for the NR_023344.1: n.43G>A variant. The mother is depicted as a circle half-filled in black, indicating carrier status for the NR_023344.1: n.43G>A variant. Segregation was confirmed through orthogonal Sanger sequencing.
(B) Illustration of key clinical features: a schematic representation of individual C1's major clinical manifestations across different life stages, from childhood through adolescence.

otherwise remain overlooked by standard global analytic tools.[5]

Conclusion

We identified bi-allelic *RNU6ATAC* variants in three individuals, guided by a distinctive transcriptomic signature of MIR in the discovery cohort and validated in an independent replication cohort. This supports *RNU6ATAC* as a disease-associated gene, defining a multisystem minor spliceopathy. Our study demonstrates that integrating transcriptomic signatures and genomic analysis offers a valuable diagnostic tool for variant pathogenicity. Ultimately, further interdisciplinary studies will be essential to fully elucidate the mechanisms of RNU6atac-opathy.

Data and code availability

All the variants in *RNU6ATAC* identified in this study were submitted to ClinVar (https://www.ncbi.nlm.nih.gov/clinvar/) (GenBank: NR_023344.2) (submitter ID 505999, UDN; submitter ID 510380, Lifera Omics). The ClinVar accession numbers of each variant are listed in Table S1.

All RNA-seq data for samples enrolled in GREGoR are available in AnVIL through dbGaP (phs003047.v3.p2). Most RNA-seq data for samples enrolled in the UDN are available through dbGaP (phs001232.v7.p3). The remaining data will be uploaded to dbGaP as a part of the next UDN data freeze. Data are also available before the next freeze by a request to the corresponding authors with evidence of dbGaP approval for UDN data.

Consortia

Members of the UDN: Alyssa A. Tran, Arjun Tarakad, Ashok Balasubramanyam, Brendan H. Lee, Carlos A. Bacino, Daryl A. Scott, Elaine Seto, Gary D. Clark, Hongzheng Dai, Hsiao-Tuan Chao, Ivan Chinn, James P. Orengo, Jill A. Rosenfeld, Kim Worley, Lindsay C. Burrage, Lisa T. Emrick, Lorraine Potocki, Monika Weisz Hubshman, Richard A. Lewis, Ronit Marom, Seema R. Lalani, Shamika Ketkar, Tiphanie P. Vogel, William J. Craigen,

Jared Sninsky, Lauren Blieden, Sandesh Nagamani, Hugo J. Bellen, Michael F. Wangler, Oguz Kanca, Shinya Yamamoto, Christine M. Eng, Patricia A. Ward, Pengfei Liu, Adeline Vanderver, Cara Skraban, Edward Behrens, Gonench Kilich, Kathleen Sullivan, Kelly Hassey, Ramakrishnan Rajagopalan, Rebecca Ganetzky, Vishnu Cuddapah, Anna Raper, Daniel J. Rader, Giorgio Sirugo, Anne Slavotinek, Christopher Mayhew, Eneida Mendonca, Ziyuan Guo, Allyn McConkie-Rosell, Kelly Schoch, Mohamad Mikati, Nicole M. Walley, Rebecca C. Spillmann, Vandana Shashi, Alan H. Beggs, Calum A. MacRae, David A. Sweetser, Deepak A. Rao, Edwin K. Silverman, Elizabeth L. Fieg, Frances High, Gerard T. Berry, Ingrid A. Holm, J. Carl Pallais, Joan M. Stoler, Joseph Loscalzo, Lance H. Rodan, Laurel A. Cobban, Lauren C. Briere, Matthew Coggins, Melissa Walker, Richard L. Maas, Susan Korrick, Jessica Douglas, Cecilia Esteves, Emily Glanton, Isaac S. Kohane, Kimberly LeBlanc, Shamil R. Sunyaev, Shilpa N. Kobren, Brett H. Graham, Erin Conboy, Francesco Vetrini, Kayla M. Treat, Khurram Liaqat, Lili Mantcheva, Stephanie M. Ware, Kathleen Page, Paul Auwaerter, Yuka Manabe, Carlos A. Pardo-Villamizar, Julie Hoover-Fong, Philip Dane Witmer, Winston Timp, Matthew Robinson, Zackary Dov Berger, Elizabeth Wohler, Nara Sobreira, Arian Nouraee, Carlos Prada, Erica Davis, Kai Lee Yap, Kelly Regan-Fendt, María Paula Silva, Patrick McMullen, Breanna Mitchell, Brendan C. Lanpher, Devin Oglesbee, Eric Klee, Filippo Pinto e Vairo, Ian R. Lanza, Kahlen Darr, Lindsay Mulvihill, Lisa Schimmenti, Queenie Tan, Surendra Dasari, Abdul Elkadri, Brett Bordini, Donald Basel, James Verbsky, Julie McCarrier, Michael Muriello, Michael T. Zimmermann, Adriana Rebelo, Carson A. Smith, Deborah Barbouth, Guney Bademci, Joanna M. Gonzalez, Kumarie Latchman, LéShon Peart, Mustafa Tekin, Nicholas Borja, Stephan Zuchner, Stephanie Bivona, Willa Thorson, Herman Taylor, Rakale C. Quarells, Ayuko Iverson, Bruce Gelb, Charlotte Cunningham-Rundles, Eric Gayle, Joanna Jen, Louise Bier, Mafalda Barbosa, Manisha Balwani, Mariya Shadrina, Rachel Evard, Saskia Shuman, Susan Shin, Vaidehi Jobanputra, Andrea Gropman, Barbara N. Pusey Swerdzewski, Camilo Toro, Colleen E. Wahl, Donna Novacic, Ellen F. Macnamara, John J. Mulvihill, Maria T. Acosta, Precilla D'Souza, Valerie V. Maduro, Ben Afzali, Ben Solomon, Cynthia J. Tifft, David R. Adams, Elizabeth A. Burke, Francis Rossignol, Heidi Wood, Jiayu Fu, Joie Davis, Leoyklang Petcharet, Lynne A. Wolfe, Margaret Delgado, Marie Morimoto, Marla Sabaii, MayChristine V. Malicdan, Neil Hanchard, Orpa Jean-Marie, Wendy Introne, William A. Gahl, Yan Huang, Andrew Stergachis, Danny E. Miller, Elisabeth Rosenthal, Elizabeth Blue, Elsa Balton, Emily Shelkowitz, Eric Allenspach, Fuki M. Hisama, Gail P. Jarvik, Ghayda Mirzaa, Ian Glass, Kathleen A. Leppig, Katrina Dipple, Mark Wener, Martha Horike-Pyne, Michael Bamshad, Peter Byers, Runjun Kumar, Seth Perlman, Sirisak Chanprasert, Virginia Sybert, Wendy Raskind, Nitsuh K. Dargie, Chun-Hung Chan, Dr. Francisco Bustos Velasq, Isum Ward, Jason Schend, Jennifer Morgan, Megan Bell, Miranda Leitheiser, Mohamad Saifeddine, Paul Berger, Rachel Li, Taylor Beagle, Alexander Miller, Beatriz Anguiano, Beth A. Martin, Brianna Tucker, Chloe M. Reuter, Devon Bonner, Elijah Kravets, Hector Rodrigo Mendez, Holly K. Tabor, Jacinda B. Sampson, Jason Hom, Jennefer N. Kohler, Jennifer Schymick, John E. Gorzynski, Jonathan A. Bernstein, Kevin S. Smith, Laura Keehan, Laurens Wiel, Matthew T. Wheeler, Meghan C. Halley, Mia Levanto, Page C. Goddard, Paul G. Fisher, Rachel A. Ungar, Raquel L. Alvarez, Sara Emami, Shruti Marwaha, Stephen B. Montgomery, Suha Bachir, Tanner D. Jensen, Taylor Maurer, Terra R. Coakley, Euan A. Ashley, Anna Hurst, Brandon M. Wilk, Bruce Korf, Elizabeth A. Worthey, Kaitlin Callaway, Martin Rodriguez, Pongtawat Lertwilaiwittaya, Reaford Blackburn, Tammi Skelton, Tarun K.K. Mamidi, Teneasha Washington, Andrew B. Crouse, Jordan Whitlock, Mariko Nakano-Okuno, Matthew Might, William E. Byrd, Albert R. La Spada, Changrui Xiao, Elizabeth C. Chao, Eric Vilain, Jose Abdenur, Kirsten Blanco, Maija-Rikka Steenari, Rebekah Barrick, Richard Chang, Sanaz Attaripour, Suzanne Sandmeyer, Tahseen Mozaffar, Alden Huang, Andres Vargas, Bianca E. Russell, Brent L. Fogel, Esteban C. Dell'Angelica, George Carvalho, Julian A. Martínez-Agosto, Layal F. Abi Farraj, Manish J. Butte, Martin G. Martin, Naghmeh Dorrani, Neil H. Parker, Rosario I. Corona, Stanley F. Nelson, Yigit Karasozen, Dana Sayer, Jennifer Tousseau, Aaron Quinlan, Alistair Ward, Ashley Andrews, Corrine K. Welt, Dave Viskochil, Erin E. Baldwin, John Carey, Justin Alvey, Lorenzo Botto, Nicola Longo, Paolo Moretti, Rebecca Overbury, Russell Butterfield, Steven Boyden, Thomas J. Nicholas, Matt Velinder, Gabor Marth, Pinar Bayrak-Toydemir, Rong Mao, Monte Westerfield, John A. Phillips III, Kimberly Ezell, Lynette Rives, Rizwan Hamid, Alyson Krokosky, Ashley McMinn, Cathy Shyr, Eric Gamazon, Joy D. Cogan, Lakshitha Perera, Lisa Bastarache, Mary Koziura, Thomas Cassini, Alex Paul, Dana Kiley, Daniel Wegner, Erin McRoy, Jennifer Wambach, Kathy Sisco, Patricia Dickson, F. Sessions Cole, Dustin Baldridge, Jimann Shin, Lilianna Solnica-Krezel, Stephen C. Pak, Timothy Schedl, Allen Bale, Carol Oladele, Caroline Hendry, Emily Wang, Hua Xu, Hui Zhang, Lauren Jeffries, María José Ortuño Romero, Mark Gerstein, Michele Spencer-Manzon, Monkol Lek, Nada Derar, Odelya Kaufman, Shrikant Mane, Teodoro Jerves Serrano, Vasilis Vasiliou, Winston Halstead, and Yong-Hui Jiang.

Members of the GREGoR consortium: Siwaar Abouhala, Sophia Adelson, Kaileigh Ahlquist, Miguel Almalvez, Emily Alsentzer, Raquel Alvarez, Mutaz Amin, Peter E. Anderson, Kailyn Anderson, Euan Ashley, Themistocles Assimes, Light Auriga, Christina Austin-Tse, Michael J. Bamshad, Rebekah Barrick, Samantha Baxter, Sairam Behera, Shaghayegh Beheshti, Gill Bejerano, Sami Belhadj, Seth Berger, Jon Bernstein, Sabrina Best, Kirsten Blanco, Benjamin Blankenmeister, Elizabeth E. Blue, Krista Bluske, Eric Boerwinkle, Emily Bonkowski, Devon Bonner, Philip M. Boone, Leandros Boukas, Denver Bradley, Harrison Brand, Kati J. Buckingham,

Daniel Calame, Colleen Carlston, Jennefer Carter, Silvia Casadei, Lisa Chadwick, Clarisa Chavez, Ziwei Chen, Allison Cheney, Yong-Han Cheng, Ivan Chinn, Jessica X. Chong, Zeynep Coban-Akdemir, Andrea J. Cohen, Sarah Conner, Matthew P. Conomos, Karen Coveler, Laura Covill, Allen Cui Ya, Colleen P. Davis, Moez Dawood, Ivan de Dios, Celine de Esch, Emmanuèle Délot, Wei Deng, Salil Deshpande, Stephanie DiTroia, Harsha Doddapaneni, Haowei Du, Michael Duyzend, Michael Duyzend, Iman Egab, Evan E. Eichler, Sara Emami, Ivy Evergreen, Mira Gandhi, Vijay Ganesh, Brandon Garcia, Kiran Garimella, Richard Gibbs, Sophia B. Gibson, Casey Gifford, Carmen Glaze, Pagé Goddard, Stephanie Gogarten, Nikhita Gogate, William W. Gordon, John E. Gorzynski, William Greenleaf, Christopher Grochowski, Emily Groopman, Rodrigo Guarischi-Sousa, Sanna Gudmundsson, (A. Gus) Gustafson Jonas, Stacey Hall, Caitlin Harrington, John Harting, William T. Harvey, Sohaib Hassan, Megan Hawley, Benjamin D. Heavner, Martha Horike-Pyne, Yun-Hua Hsiao, Jianhong Hu, Yongqing Huang, Karan Jaisingh, Minal Jamsandekar, Gail P. Jarvik, Tanner Jensen, Shalini Jhangiani, David Jimenez-Morales, Christopher Jin, Aimee Juan, Ahmed K. Saad, Jessica Kain, Rachid Karam, Laura Keehan, Sky Kim, Hadley King Charles, Julia Klugherz, Arthur Ko, Anshul Kundaje, Soumya Kundu, Samuel M. Lancaster, Katie Larsson, Arthur Lee, Gabrielle Lemire, Mia Levanto, Jesse Levine, Wei Li, Pengfei Liu, Bojan Losic, Jonathan LoTempio, James (Jim) Lupski, Jialan Ma, Daniel MacArthur, Annelise Y. Mah-Som, Medhat Mahmoud, Brian Mangilog, Dana Marafi, Daniel Marten, Eva Martinez, Colby T. Marvin, Shruti Marwaha, F. Kumara Mastrorosa, Dena Matalon, Taylor Maurer, Susanne May, Sean R. McGee, Lauren Meador, Heather C Mefford., Rodrigo Mendez Hector, Olfa Messaoud, Alexander Miller, Danny E. Miller, Romal Mitr, Stephen Montgomery, Yulia Mostovoy, Mariana Moyses, Chloe Munderloh, Donna Muzny, Ashana Neale, Sarah C. Nelson, Matthew B. Neu, Jonathan Nguyen, Thuy-mi P. Nguyen, Annie Niehaus, Robert Nussbaum, Emily O'Heir, Briana O'Leary, Melanie O'Leary, Sebastian Ochoa Gonzalez, Jeren Olsen, Osei-Owusu Ikeoluwa, Anne O'Donnell-Luria, Miranda P.G. Zalusky, Evin Padhi, Lynn Pais, Piyush Panchal, Shruti Pande, Karynne E. Patterson, Sheryl Payne, Davut Pehlivan, Paul Petrowski, Alicia Pham, Georgia Pitsava, Astaria (Sara) Podesta, Elizabeth Porter, Jennifer Posey, Jaime Prosser, Guanghao Qi, Wanqiong Qiao, Thomas Quertermous, Archana Rai, Heidi Rehm, Chloe Reuter, Matthew A. Richardson, Andres Rivera-Munoz, Lindsay Romo, Oriane Rubio, Kathryn Russell, Aniko Sabo, Ismail Safi, Monica Salani, Kaitlin Samocha, Armando Sanchez-Conde, Alba Sanchis-Juan, Sarah Savage, Jacob Schmidt, Evette Scott, Stuart Scott, Adriana E. Sedeño-Cortés, Fritz Sedlazeck, Jillian Serrano, Gulalai Shah, Deepali Shinde, Ali Shojaie, Moriel Singer-Berk, Mugdha Singh, Riya Sinha, Joshua D. Smith, Kevin Smith, Hana Snow, Michael Snyder, Kayla Socarras, Olivia M. Sommerland, Lea M. Starita, Brigitte Stark, Sarah Stenton, Andrew B. Stergachis, Adrienne Stilp, V. Reid Sutton, Elliott G. Swanson, Jui-Cheng Tai, Michael Talkowski, Christina G.Tise, Catherine C. Tong, Philip Tsao, Rachel Ungar, Grace VanNoy, Eric Vilain, Gaby Villard, Mitchell R. Vollger, Isabella Voutos, Kim Walker, Juliana Walrod, Chia-Lin Wei, Ben Weisburd, Jeffrey M. Weiss, Chris Wellington, Ziming Weng, Lauren Westerfield, Matthew Wheeler, Marsha Wheeler, Laurens Wiel, Michael Wilson, Monica Wojcik, Chee Hong Wong, Issac Wong, Quenna Wong, Frank Wong, Changrui Xiao, Jiaoyang Xu, Rachita Yadav, Yao Yang, Qian Yi, Jiye Yu, Bo Yuan, Christina Zakarian, Jimmy Zhen.

Acknowledgments

We thank all research individuals in the UDN and GREGoR consortium. We would also like to thank members of the Wheeler lab, the Montgomery lab, the GREGoR consortium, the UDN, and the Stanford Center for Undiagnosed Diseases, who gave invaluable feedback and assistance throughout this project. Figures were created in BioRender (https://BioRender.com/emhjjfv). This work required computing resources from the Stanford Genetics Bioinformatics Service Center (supported by NIH Instrumentation grant S10 OD025082). The work would not have been possible without the Stanford SCG cluster administrators, specifically Chris Jeon and Karl Kornel. R.J.L. was supported by CNCDP-K12 funding. T.M.A. was supported by the National Science Foundation Graduate Research Fellowship under grant no. DGE-2146755. Research reported in this paper was partly funded by the National Human Genome Research Institute at the National Institutes of Health, United States, as part of the GREGoR consortium, through grant nos. U01HG011762 and U01HG011755. This publication was also supported by the National Institute of Neurological Disorders and Stroke of the National Institutes of Health under award number U01NS134358. The content is solely the responsibility of the authors and does not necessarily represent the official views of the National Institutes of Health.

Author contributions

Project conceptualization, V.S.G., A.O.L., J.A.B., S.B.M., and M.T.W.; ethics approval, D.E.B.; participant recruitment and clinical data contribution, S.E., R.J.L., D.N., R.M., D.E.B., F.S.A., A.A., B.A., and K.B.; data generation, K.S.S., S.A.S., L.L., Z.N., F.S.A., and K.B.; data analysis, T.M.A., V.S.G., J.M., R.M., R.A.U., A.M.M., J.N., S.M., and A.W.; figure generation, R.M., T.M.A., and D.E.B.; writing – original draft, R.M., T.M.A., and D.E.B.; writing – review & editing, all authors.

Declaration of interests

S.B.M. is a member of the scientific advisory boards of MyOme, PhiTech, and Valinor Therapeutics. A.W. is chief executive officer of Frameshift Labs (developer of Mosaic). R.J.L. is an unpaid member of the scientific advisory boards for the Timothy Syndrome Foundation and the Timothy Syndrome Alliance.

Supplemental information

Supplemental information can be found online at https://doi.org/10.1016/j.xhgg.2026.100588.

Web resources

ClinVar, https://www.ncbi.nlm.nih.gov/clinvar/

Online Mendelian Inheritance in Man (OMIM), http://www.omim.org

UCSC Genome Browser[16] "Exome Probesets" track (GRCh38/hg38), http://genome.ucsc.edu

Received: November 19, 2025
Accepted: March 3, 2026

References

1. Olthof, A.M., Hyatt, K.C., and Kanadia, R.N. (2019). Minor intron splicing revisited: identification of new minor intron-containing genes and tissue-dependent retention and alternative splicing of minor introns. BMC Genom. *20*, 686. https://doi.org/10.1186/s12864-019-6046-x.
2. Zhao, J., Peter, D., Brandina, I., Liu, X., and Galej, W.P. (2025). Structural basis of 5′ splice site recognition by the minor spliceosome. Mol. Cell *85*, 652–664.e4.
3. Schneider, C., Will, C.L., Makarova, O.V., Makarov, E.M., and Lührmann, R. (2002). Human U4/U6.U5 and U4atac/U6atac.U5 tri-snRNPs exhibit similar protein compositions. Mol. Cell Biol. *22*, 3219–3229. https://doi.org/10.1128/MCB.22.10.3219-3229.2002.
4. Ciavarella, J., Perea, W., and Greenbaum, N.L. (2020). Topology of the U12-U6atac snRNA complex of the minor spliceosome and binding by NTC-related protein RBM22 ACS Omega *5*, 23549–23558. https://doi.org/10.1021/acsomega.0c01674.
5. Arriaga, T.M., Mendez, R., Ungar, R.A., Bonner, D.E., Matalon, D.R., Lemire, G., Goddard, P.C., Padhi, E.M., Miller, A.M., Nguyen, J.V., et al. (2025). Transcriptome-wide outlier approach identifies individuals with minor spliceopathies. Am. J. Hum. Genet. *112*, 2458–2475. https://doi.org/10.1016/j.ajhg.2025.08.018.
6. Bai, R., Wan, R., Wang, L., Xu, K., Zhang, Q., Lei, J., and Shi, Y. (2021). Structure of the activated human minor spliceosome. Science *371*, eabg0879. https://doi.org/10.1126/science.abg0879.
7. Antonarakis, S.E. (2026). Small nuclear RNA genes in Mendelian disorders. Nat. Genet. *58*, 28–38. https://doi.org/10.1038/s41588-025-02440-7.
8. Merico, D., Roifman, M., Braunschweig, U., Yuen, R.K.C., Alexandrova, R., Bates, A., Reid, B., Nalpathamkalam, T., Wang, Z., Thiruvahindrapuram, B., et al. (2015). Compound heterozygous mutations in the noncoding RNU4ATAC cause Roifman Syndrome by disrupting minor intron splicing. Nat. Commun. *6*, 8718. https://doi.org/10.1038/ncomms9718.
9. Elsaid, M.F., Chalhoub, N., Ben-Omran, T., Kumar, P., Kamel, H., Ibrahim, K., Mohamoud, Y., Al-Dous, E., Al-Azwani, I., Malek, J.A., et al. (2017). Mutation in noncoding RNA RNU12 causes early onset cerebellar ataxia. Ann. Neurol. *81*, 68–78.
10. Benoit-Pilven, C., Besson, A., Putoux, A., Benetollo, C., Saccaro, C., Guguin, J., Sala, G., Cologne, A., Delous, M., Lesca, G., et al. (2020). Clinical interpretation of variants identified in RNU4ATAC, a non-coding spliceosomal gene. PLoS One *15*, e0235655.
11. He, H., Liyanarachchi, S., Akagi, K., Nagy, R., Li, J., Dietrich, R.C., Li, W., Sebastian, N., Wen, B., Xin, B., et al. (2011). Mutations in U4atac snRNA, a Component of the Minor Spliceosome, in the Developmental Disorder MOPD I. Science *332*, 238–240.
12. Xing, C., Kanchwala, M., Rios, J.J., Hyatt, T., Wang, R.C., Tran, A., Dougherty, I., Tovar-Garza, A., Purnadi, C., Kumar, M.G., et al. (2021). Biallelic variants in RNU12 cause CDAGS syndrome. Hum. Mutat. *42*, 1042–1052.
13. Ungar, R.A., Goddard, P.C., Jensen, T.D., Degalez, F., Smith, K.S., Jin, C.A., Undiagnosed Diseases Network, Bonner, D.E., Bernstein, J.A., Wheeler, M.T., and Montgomery, S.B. (2024). Impact of genome build on RNA-seq interpretation and diagnostics. Am. J. Hum. Genet. *111*, 1282–1300.
14. Turunen, J.J., Niemelä, E.H., Verma, B., and Frilander, M.J. (2013). The significant other: splicing by the minor spliceosome. Wiley Interdiscip. Rev. RNA *4*, 61–76.
15. Mertes, C., Scheller, I.F., Yépez, V.A., Çelik, M.H., Liang, Y., Kremer, L.S., Gusic, M., Prokisch, H., and Gagneur, J. (2021). Detection of aberrant splicing events in RNA-seq data using FRASER. Nat. Commun. *12*, 529.
16. Raney, B.J., Barber, G.P., Benet-Pagès, A., Casper, J., Clawson, H., Cline, M.S., Diekhans, M., Fischer, C., Navarro Gonzalez, J., Hickey, G., et al. (2024). The UCSC Genome Browser database: 2024 update. Nucleic Acids Res. *52*, D1082–D1088. https://doi.org/10.1093/nar/gkad987.
17. Karczewski, K.J., Francioli, L.C., Tiao, G., Cummings, B.B., Alföldi, J., Wang, Q., Collins, R.L., Laricchia, K.M., Ganna, A., Birnbaum, D.P., et al. (2020). The mutational constraint spectrum quantified from variation in 141,456 humans. Nature *581*, 434–443.
18. Chen, S., Francioli, L.C., Goodrich, J.K., Collins, R.L., Kanai, M., Wang, Q., Alföldi, J., Watts, N.A., Vittal, C., Gauthier, L.D., et al. (2024). A genomic mutational constraint map using variation in 76,156 human genomes. Nature *625*, 92–100. https://doi.org/10.1038/s41586-023-06045-0.
19. Landrum, M.J., Lee, J.M., Benson, M., Brown, G.R., Chao, C., Chitipiralla, S., Gu, B., Hart, J., Hoffman, D., Jang, W., et al. (2018). ClinVar: improving access to variant interpretations and supporting evidence. Nucleic Acids Res. *46*, D1062–D1067. https://doi.org/10.1093/nar/gkx1153.
20. Bai, R., Yuan, M., Zhang, P., Luo, T., Shi, Y., and Wan, R. (2024). Structural basis of U12-type intron engagement by the fully assembled human minor spliceosome. Science *383*, 1245–1252. https://doi.org/10.1126/science.adn7272.
21. Schubach, M., Maass, T., Nazaretyan, L., Röner, S., and Kircher, M. (2024). CADD v1.7: using protein language models, regulatory CNNs and other nucleotide-level scores to improve genome-wide variant predictions. Nucleic Acids Res. *52*, D1143–D1154. https://doi.org/10.1093/nar/gkad989.
22. Tenywa, J.F., Lamouche, J.B., Baer, S., Nicaise, S., Le Béchec, A., Piton, A., and Muller, J. (2025). Genome region aware CADD thresholds for noncoding variant prioritization. NAR Genom. Bioinform. *7*, lqaf157. https://doi.org/10.1093/nargab/lqaf157.
23. Sobreira, N., Schiettecatte, F., Valle, D., and Hamosh, A. (2015). GeneMatcher: a matching tool for connecting investigators with an interest in the same gene. Hum. Mutat. *36*, 928–930. https://doi.org/10.1002/humu.22844.
24. Verma, B., Akinyi, M.V., Norppa, A.J., and Frilander, M.J. (2018). Minor spliceosome and disease. Semin. Cell Dev. Biol. *79*, 103–112.

25. Farach, L.S., Little, M.E., Duker, A.L., Logan, C.V., Jackson, A., Hecht, J.T., and Bober, M. (2018). The expanding phenotype of RNU4ATAC pathogenic variants to Lowry Wood syndrome. Am. J. Med. Genet. *176*, 465–469. https://doi.org/10.1002/ajmg.a.38581.

26. Heremans, J., Garcia-Perez, J.E., Turro, E., Schlenner, S.M., Casteels, I., Collin, R., de Zegher, F., Greene, D., Humblet-Baron, S., Lesage, S., et al. (2018). Abnormal differentiation of B cells and megakaryocytes in patients with Roifman syndrome. J. Allergy Clin. Immunol. *142*, 630–646. https://doi.org/10.1016/j.jaci.2017.11.061.

27. Cologne, A., Benoit-Pilven, C., Besson, A., Putoux, A., Campan-Fournier, A., Bober, M.B., De Die-Smulders, C.E.M., Paulussen, A.D.C., Pinson, L., Toutain, A., et al. (2019). New insights into minor splicing-a transcriptomic analysis of cells derived from TALS patients. RNA *25*, 1130–1149. https://doi.org/10.1261/rna.071423.119.

HGGA, Volume 7

Supplemental information

Bi-allelic *RNU6ATAC* variants cause a minor spliceopathy characterized by transcriptome-wide minor intron retention and multisystem manifestations

Rodrigo Mendez, Taylor M. Arriaga, Jialan Ma, Devon E. Bonner, Sara Emami, Rebecca J. Levy, Afaf Alsagheir, Bader Alhaddad, Khadijah Bakur, Rachel A. Ungar, Dena R. Matalon, Alexander M. Miller, Jonathan Nguyen, Kevin S. Smith, Stuart A. Scott, Linda Liao, Zena Ng, Shruti Marwaha, Alistair Ward, Undiagnosed Diseases Network, Genomics Research to Elucidate the Genetics of Rare Diseases Consortium, Danica Novacic, Fowzan S. Alkuraya, Jonathan A. Bernstein, Vijay S. Ganesh, Anne O'Donnell-Luria, Stephen B. Montgomery, and Matthew T. Wheeler

Supplemental Material 2

Clinical Vignettes and Genetic Findings for RNU6atac-opathy

Individual A1 [NR_023344.1: n.28C>T; NR_023344.1:n.36T>G]; one case

Individual A1 is a 14-year-old female with a multisystem neurodevelopmental phenotype, an unremarkable family history, and a healthy sister (Figure 2A). Prenatally, intrauterine growth restriction was noted. Birth weight was 2.3 kg [Z = -2.03], length was 48.3 cm [Z = -0.41], and head circumference was 33 cm [Z = -1.11]. She experienced refractory epilepsy with seizures beginning on day three of life and was hospitalized in the neonatal intensive care unit until day twenty-three of life. She was noted to have bilateral 2-3 toe syndactyly. She was readmitted soon after discharge for failure to thrive and hyperbilirubinemia. Her infantile history was notable for poor feeding with global growth restriction and early onset nystagmus at 5 months old. She had a recurrence of epilepsy at 8 months old, with focal temporal status epilepticus. A brain MRI at that time revealed cerebral underdevelopment, characterized by a relatively small genu of the corpus callosum, moderate enlargement of the lateral ventricles, and mild enlargement of the third ventricle, as well as absence of the left 6th cranial nerve in the expected location. Given a clinical exam with limited but not absent left eye abduction, that cranial nerve is presumed to be in an atypical location. Since then, she has had treatment-refractory epilepsy. She walked at 2.5 years; her first word was at 2 years. Currently, her speech consists of occasional spontaneous one- to two-word utterances, with a total spontaneous vocabulary of about five words, although she exhibits echolalia and repeats long phrases. She was diagnosed with autism and severe intellectual disability. She developed episodic ataxia at age three, which became persistent by early childhood with a wide-based gait. Ophthalmological findings included strabismus, oculomotor apraxia, and hyperopic astigmatism. She has experienced heavy menstrual bleeding since menarche, with coagulation studies demonstrating a prolonged prothrombin time. Cardiovascular evaluation revealed a heart murmur that resolved in childhood (no echocardiogram was performed), with an ECG showing a sinus rhythm and nonspecific intraventricular conduction delay. Gastrointestinal issues included chronic constipation, abdominal pain, and dysmotility (Figure 2B). Clinical genetics evaluation included chromosomal

microarray analysis, chromosomal breakage analysis, mtDNA testing, trio genome sequencing, and biochemical testing (plasma amino acids, urine organic acids, acylcarnitines, very long-chain fatty acids, and carnitine). Creatine kinase was mildly elevated at 2 years (224 U/L; ref <150) but later normalized. At her last follow-up at 13 years and 7 months old, her height was 141 cm [Z = -2.77], her weight was 37.7 kg [Z = -1.45], and her head circumference was 49 cm [Z < -2.05]. She had new flexural eczema involving the knees and elbows; the skin exam showed generalized xerosis with scattered superficial healing excoriations on the back and limbs, and a few round hyperpigmented leg patches. Laboratory testing documented marked peripheral eosinophilia with an absolute eosinophil count of 10,000/µL.

Genetic findings: Individual A1 had compound heterozygous *RNU6ATAC* (NR_023344.1) variants (The NR_023344.1: n.28C>T, maternal inheritance; The NR_023344.1: n.36T>G, paternal inheritance). The n.28C>T variant (gnomAD v4[1,2] allele frequency: 7.9×10^{-5}; CADD[3]: 21; PhyloP100[4]: 9.55) affects a nucleotide that participates in base pairing within the Stem I region of U4atac that is crucial for splicing[5]. The n.36T>G variant, absent from gnomAD v4.1.0[1,2] (CADD[3]: 18; PhyloP100[4]: 7.12)[5], disrupts a conserved uridine in the U6atac stem II region, potentially destabilizing its structure[5] (Figure 1D). Sanger sequencing confirmed that the proband's sister did not carry either of the identified *RNU6ATAC* variants.

Individual B1 [NR_023344.1: n.30C>T; NR_023344.1: n.64C>G]; one case

Individual B1 was a 30-year-old male with a multisystem disorder characterized by prominent immune dysfunction, endocrinopathy, and ectodermal abnormalities. Family history was unremarkable, with no reported consanguinity (Figure 3A). He was born at term to a 32-year-old mother after an uncomplicated pregnancy and spontaneous vaginal delivery. Infancy was notable for primary hypothyroidism and failure to thrive; he had hypogammaglobulinemia and recurrent sinopulmonary infections. Immunological evaluation revealed a phenotype of Combined Variable Immunodeficiency (CVID) manifested by low serum immunoglobulins (IgA and IgG) and, crucially, severely reduced counts of CD4+ T cell and B cell subsets (detailed immunophenotyping, see **Supplemental Material 3**).

At age five, persistent gastrointestinal symptoms prompted evaluation, revealing eosinophilic colitis and exocrine pancreatic insufficiency with chronic steatorrhea and low stool elastase, which was long-term treated with pancrelipase with good response. Protein-losing enteropathy

and malabsorption were documented. Growth parameters remained below the third percentile throughout childhood and adolescence, and in adulthood, his height and weight were 165 cm [Z < -1.61] and 42.7 kg [Z < -3.89] (body mass index approximately 15.7 kg/m²), consistent with chronic growth failure. From early childhood, he had ichthyosis, dystrophic nails, and alopecia universalis. Dental anomalies included peg-shaped teeth with irregular spacing and cupping. During adolescence, episodes of acute muscle weakness and areflexia led to a diagnosis of chronic inflammatory demyelinating polyneuropathy (CIDP); scoliosis developed, and later contractures and sarcopenia. Respiratory disease evolved with asthma, recurrent pneumonias, and chest imaging identified bronchiectasis. Despite medical complexity, he achieved typical developmental milestones and completed high school; he initiated but withdrew from community college during the first year. He had no specific learning disability, although frequent illness-related absences and suboptimal adherence to thyroid replacement impacted schooling. (Figure 3B). Given this constellation of features, he initially received a clinical diagnosis of APECED (Autoimmune Polyglandular Syndrome Type 1 [MIM: 240300]); however, AIRE (autoimmune regulator [MIM: 607358]) gene sequencing was normal. Additional genetic evaluations included trio clinical genome sequencing, which was non-diagnostic. Ultimately, in the setting of bronchiectasis and neuromuscular weakness, he developed respiratory failure requiring intubation and could not be weaned; tracheostomy was declined, and death occurred at age 30 years.

Genetic findings: Individual B1 harbored two *RNU6ATAC* variants in trans, NR_023344.1: n.30C>T, inherited from the mother, and NR_023344.1: n.64C>G inherited from the father. The NR_023344.1: n.30C>T variant (gnomAD v4.1.0[1,2] allele frequency: 6.6×10^{-6}; CADD[3]: 21; PhyloP100[4]: 7.62) is located at the Stem I/II boundary of the U4atac/U6atac bimolecule, which is essential for activation of the spliceosome before catalysis and is reported in ClinVar[6] as of uncertain significance (RCV004764396.1). The NR_023344.1: n.64C>G variant, absent from gnomAD v4.1.0[1,2] (CADD[3]: 19; PhyloP100[4]: 3.98), disrupts the distinctive central Stem-loop of U6atac. The central stem-loop is known [1,2]to be stabilized by specific protein-RNA interactions with CENATAC (centrosomal AT-AC splicing factor [MIM: 6200142]), which neutralize the RNA's negative charge. Disruptions in this region could destabilize the U6atac structure, adversely affecting minor intron splicing[7].

Individual C1 [NR_023344.1: n.43G>A, homozygous]; one case

Individual C1 is a 17-year-old male born to first-cousin parents; he also has two healthy older sisters. He was born at 32 weeks' gestation via induced vaginal delivery due to decreased fetal movements and poor fetal growth. His birth weight was 1.5 kg [Z = -1.6]; other birth parameters were not available. He required neonatal intensive care for three weeks for growth support.

The participant presented at 7 months old with a history of recurrent scalp abscesses and chest infections starting at 2 months. While the complete blood count was unremarkable: WBC 9.6 × 10^9/L, hemoglobin 118 g/L, platelets 331 × 10^9/L, ANC 3.25 × 10^9/L, ALC 4.18 × 10^9/L, immunological evaluation identified profound pan-hypogammaglobulinemia. IgG was markedly reduced at 310 mg/dL (reference range: 660–1530 mg/dL), with IgA <25 mg/dL (reference: 70–400 mg/dL) and IgM <21 mg/dL (reference: 25–259 mg/dL); IgE was 22.2 UI/mL. Lymphocyte immunophenotyping revealed B-cell lymphopenia (CD19: 238 cells/µL), whereas T-cell and NK-cell counts were preserved (CD3: 2773, CD4: 2400, CD8: 515, NK: 336 cells/µL). Despite the antibody deficiency, T-cell function appeared intact, as evidenced by normal lymphocyte responses to mitogens: Phytohemagglutinin (PHA) 260,148 CPM; Concanavalin-A (Con-A) 81,255 CPM; Pokeweed 78,394 CPM; and Pooled Allogeneic Cells 36,466 CPM. Furthermore, expression of CD40 and CD40 ligand was intact, and oxidative burst testing by flow cytometry was normal (87% of PMNs reduced DHR-123 post-PMA stimulation).

He was diagnosed with antibody deficiency and commenced on immunoglobulin replacement therapy (IVIG, later subcutaneous), which he continues to receive. Despite treatment, he suffered from recurrent lower respiratory tract infections leading to progressive lung damage. Chest CT at age 9 confirmed advanced bilateral bronchiectasis with right middle lobe obstruction.

He exhibits a complex endocrine phenotype characterized by early-onset Type 1 diabetes mellitus (diagnosed at 9 months) and acquired hypothyroidism requiring levothyroxine. He also has a history of cryptorchidism requiring orchidopexy. Postnatal growth has been severely affected; he developed microcephaly and significant short stature. Growth hormone levels were normal, and a therapeutic trial of growth hormone yielded no improvement.

Skeletal survey revealed epiphyseal dysplasia. He developed progressive lower limb deformities, specifically severe bilateral genu valgum necessitating multiple surgical interventions, including hemiepiphysiodesis at age 8 and subsequent corrective osteotomies with ligament reconstruction. However, surgical outcomes were poor, with persistent angular deformity, bilateral fixed flexion

contractures, and global knee instability (including lateral patellar dislocation and 90-degree tibial external rotation). He is currently wheelchair-bound.

Global developmental delay was noted in infancy; he sat unsupported at 1 year and walked at 3 years. He exhibits severe delays in receptive and expressive language. Cognitive testing (Beery VMI) at age 7 showed a standard score of 70 (age equivalent: 55 months). Sensory evaluation revealed progressive retinal dystrophy characterized by nyctalopia, flat visual evoked potentials (VEP), and loss of outer retinal segments on Optical coherence tomography (OCT). Additionally, although hearing was normal in early childhood, reassessment at age 15 revealed bilateral high-frequency sensorineural hearing loss (mildly sloping to moderately severe). Dental anomalies include a congenitally missing lower right second premolar.

Genetic findings: Individual C1 was found to be homozygous for the *RNU6ATAC* variant NR_023344.1:n.43G>A, both parents were confirmed carriers, while his two healthy sisters were not genotyped. This variant is rare (allele frequency: 0.00001313), being absent in homozygous states in gnomAD v4.1.0[1,2] dataset. In silico analysis supports a deleterious effect (CADD[3]: 19; PhyloP100[4]: 4.72). Structurally, the n.43G>A variant maps to Stem II of the U4atac/U6atac bimolecule, a region critical for the assembly of the di-snRNP complex. The functional importance of this nucleotide is underscored by its structural partner: *RNU6ATAC* n.43 base-pairs with nucleotide n.8 of *RNU4ATAC*. The corresponding *RNU4ATAC* n.8 position is a recognized mutational hotspot[8], with multiple substitutions (n.8C>A, n.8C>T, and n.8C>G) reported as pathogenic or likely pathogenic in ClinVar[6]. This suggests that disrupting the Watson-Crick base pairing at this specific site within Stem II destabilizes the U4atac/U6atac complex, impairing minor spliceosome function.

References

1. Karczewski KJ, Francioli LC, Tiao G, Cummings BB, Alföldi J, Wang Q, et al. The mutational constraint spectrum quantified from variation in 141,456 humans. Nature [Internet]. 2020 May;581(7809):434–43. Available from: http://dx.doi.org/10.1038/s41586-020-2308-7

2. Chen S, Francioli LC, Goodrich JK, Collins RL, Kanai M, Wang Q, et al. A genomic mutational constraint map using variation in 76,156 human genomes. Nature [Internet]. 2024 Jan;625(7993):92–100. Available from: http://dx.doi.org/10.1038/s41586-023-06045-0

3. Schubach M, Maass T, Nazaretyan L, Röner S, Kircher M. CADD v1.7: using protein language models, regulatory CNNs and other nucleotide-level scores to improve genome-wide variant predictions. Nucleic Acids Res [Internet]. 2024 Jan 5;52(D1):D1143–54. Available from: http://dx.doi.org/10.1093/nar/gkad989

4. Raney BJ, Barber GP, Benet-Pagès A, Casper J, Clawson H, Cline MS, et al. The UCSC Genome Browser database: 2024 update. Nucleic Acids Res [Internet]. 2024 Jan 5;52(D1):D1082–8. Available from: http://dx.doi.org/10.1093/nar/gkad987

5. Arriaga MT, Mendez R, Ungar RA, Bonner DE, Matalon DR, Lemire G, et al. Transcriptome-wide outlier approach identifies individuals with minor spliceopathies. medRxiv [Internet]. 2025 Jan 3;2025.01.02.24318941. Available from: http://dx.doi.org/10.1101/2025.01.02.24318941

6. Landrum MJ, Lee JM, Benson M, Brown GR, Chao C, Chitipiralla S, et al. ClinVar: improving access to variant interpretations and supporting evidence. Nucleic Acids Res [Internet]. 2018 Jan 4;46(D1):D1062–7. Available from: http://dx.doi.org/10.1093/nar/gkx1153

7. Bai R, Yuan M, Zhang P, Luo T, Shi Y, Wan R. Structural basis of U12-type intron engagement by the fully assembled human minor spliceosome. Science [Internet]. 2024 Mar 15;383(6688):1245–52. Available from: http://dx.doi.org/10.1126/science.adn7272

8. Benoit-Pilven C, Besson A, Putoux A, Benetollo C, Saccaro C, Guguin J, et al. Clinical interpretation of variants identified in RNU4ATAC, a non-coding spliceosomal gene. PLoS One [Internet]. 2020 Jul 6 [cited 2025 Mar 14];15(7):e0235655. Available from: https://journals.plos.org/plosone/article?id=10.1371/journal.pone.0235655

Immunophenotype Characterization of Individual B1.

Individual B1 had significant immune dysfunction and initially received a clinical diagnosis of APECED (Autoimmune Polyglandular Syndrome Candidiasis and Ectodermal Dysplasia), also known as autoimmune polyglandular syndrome type-1 (APS1 [MIM: 240300]). However, *AIRE* (autoimmune regulator [MIM: 607358]) gene sequencing was normal, and several features did not fully align with the typical autoimmune dysfunction of APECED, including the absence of aberrant autoantibodies and candidal infections.

He did have Combined Variable Immune Deficiency (CVID) manifested as low immunoglobulins IgA and IgG. He had recurrent sinopulmonary infections, including pneumonia, requiring hospitalization and residual chronic lung sequela of a 2.2cm cavitary lesion in the left lower lobe, bilateral bronchiectasis, tree-in-bud, as well as ground-glass opacities. Sweat chloride was 35 mmol/L, then repeated at 36 mmol/L, which is intermediate. He had chronic protein-losing enteropathy along with eosinophilia on gastrointestinal biopsies, but IgE was normal. Peripheral blood eosinophils were mildly elevated at 890 K/ul (normal 40-540 K/uL). He was never septic. He had poor responses to some vaccines, including Pneumococcal, Hepatitis B, and Polio. Rubella titer was protective. Hemophilus influenzae titer was suboptimal at 0.17 ug/mL (short-term protection >0.15 ug/mL, long-term protective >1.0 ug/mL), and he subsequently developed H. influenzae pneumonia, confirmed by bronchoalveolar lavage samples. We did not find evidence of aberrant antibody production, including negative autoimmune antibodies for Lupus panel, Celiac, and thyroid autoantibodies. T cell mitogen-stimulation responses were normal, suggesting normal CD8 T Cell function, including a normal response to Candida antigen. On immune phenotyping, CD4 memory subsets and B cells were low. During adolescence, he developed muscle weakness and areflexia and was found to have a demyelinating polyneuropathy on nerve conduction testing. This was diagnosed as Chronic Inflammatory Demyelinating Polyneuropathy (CIDP). No known CIDP auto-antibodies were found. This was still presumed to be autoimmune, possibly CD4 T cell-mediated. Ultimately,

from CIDP, he developed muscle contractures and respiratory muscle weakness. He was treated with Intravenous Gammaglobulin. This did help prevent recurrent infections, but CIDP still progressed. Overall, his hypogammaglobulinemia may have been secondary to reduced B cell subsets due to poor/abnormal helper CD4 T cell stimulation. Low protein stores from chronic GI losses could have also played a role in his hypogammaglobulinemia. On peripheral blood flow, there was also a curious population of double-positive T cells (CD4+/CD8+) at 4.2% (normal <0.1%) of unclear significance, and CD4 memory cells were mildly low. We speculated that, together, these manifestations may point to a primary problem in proper CD4 T cell development in the thymus, including possibly poor interactions between thymic epithelial cells and maturing T cells, especially given the similarity of his phenotype to APECED. Further investigation of thymic function was not possible clinically.

Table: Notable immune labs

**Peripheral blood lab**	**Result**	**Normal Range**
**CBC (complete blood counts)**		
White blood cells total	6.14	4.23-9.07 K/uL
Lymphocytes total %	10.8	21.8-53.1%
Lymphocytes #	0.66	1.32-3.57 K/uL
**Lymphocyte phenotypes (primary immunodeficiency flow panel)**		
T4/T8 Ratio	1.39	1.11-5.17 Ratio
CD4 %	46.1	31.9-62.2 %
CD4 #	304	359-1565 /uL
CD8 %	33.1	11.2-34.8 %
CD8 #	218	178-853 /uL
CD4+/CD8+ %	4.2	<0.1%
CD4+/CD8+ #	28	0 /uL
CD4+/CD62L+/CD45RA- % (CD4 T central memory)	20.7	10.4-30.7 %

CD4+/CD62L+/CD45RA- #	137	162-614 /uL
CD4+/CD62L-/CD45RA- % (CD4 T peripheral memory)	1.6	2.3-15.6 %
CD4+/CD62L-/CD45RA- #	11	42-225 /uL
CD20 % (B cells)	0.3	3.0-19.0 %
CD20 #	2	59-329 /uL
CD19 %	0.6	3.3-19.3 %
CD19 #	4	61-321 /uL
Other subsets normal or not substantially skewed:		
NK		
NK/T		
DNT (double negative T)		
CD4 central naive		
**Immunoglobulins**		
IgG (pre-IV Ig treatment)	322.0	700-1600 mg/dL
IgA	45.0	70-400 mg/dL
IgM	48.0	40-230 mg/dL
IgE	2.4	0.0-90.0 IU/mL

Genome Sequencing

Whole Genome Sequencing (WGS) for participants A1 and B1, as well as their respective family members, was performed by Baylor Genetics, through the Undiagnosed Disease Network (UDN), using methods previously described by Splinter et al., 2018[1]. In brief, libraries were prepared using a PCR-free 550-bp insert size protocol by the Hyper Prep kit. Sequencing was performed using the Illumina NovaSeq 6000 platform for 150 bp paired-end reads. The SNPTrace Panel from the Fluidigm SNPtype platform was applied as a quality control measure. The Illumina Dragen BioIT Platform performed data analysis and interpretation. The FASTQ data were aligned to the human reference genome build GRCh38 using the Illumina Dragen BioIT Platform. Additionally, we used the Illumina Dragen haplotype-based variant calling system to perform variant calling on all resulting BAM files.

Clinical Genome Sequencing (CGS) was performed for individual C1 and his parents at Centogene. Genomic DNA was extracted from blood samples (CentoCards) using the QIASymphony magnetic bead–based method (Qiagen). DNA was fragmented by sonication, ligated to Illumina adapters, and sequenced on the Illumina HiSeq X platform, achieving >30X average genome coverage. Sequence data were aligned to the hg19 (GRCh37) reference genome. Variants were called and annotated using a validated in-house pipeline. SNVs and small indels were filtered using a semi-automated strategy, and variants with insufficient quality scores were confirmed by Sanger sequencing.

RNA-seq library preparation and sequencing

We performed RNA-sequencing on 422 whole blood samples and 139 fibroblast samples from the Genomics Research to Elucidate the Genetics of Rare diseases (GREGoR) and UDN consortia, as well as the Broad Center for Mendelian Genomics (Broad CMG) and self-funded individuals. All 422 whole blood samples were previously published in Arriaga et al., 2025[2], 287

of which were also published in Ungar et al., 2024[3]. The fibroblast cohort included samples previously published by Cummings et al., 2017[4]. Ethical and research approvals were provided by the Stanford University IRB (protocol 60837) and the National Human Genome Research Institute Institutional Review Board (IRB) (protocol 15-HG-0130, protocol 2013P001477). All participants provided informed consent.

The experimental protocol and computational pipeline for 287 of the 422 whole blood samples are described in Ungar et al., 2024[3]. Of these, eight samples were collected and processed in PAXgene tubes at the Utah UDN site before being shipped to Stanford. In brief, cDNA libraries were generated using either the Illumina TruSeq Stranded mRNA Sample Prep Kit protocol and dual-indexed, or the Universal Plus mRNA-seq NuQuant library prep protocol from Tecan, following the same protocol as Amar et al., 2024[5].

The experimental methods for the remaining 135 whole blood samples, seven of which were collected and processed at the Miami UDN site, are described in Arriaga et al., 2025[2]. In short, at Stanford, the cDNA libraries were generated using a Biomek i7 Liquid Handler robot with Tecan library-specific scripts, and pooled libraries were sequenced as 2x150bp paired-end reads on an Illumina Novaseq S2 Flow Cell. At the Miami UDN site, cDNA libraries were generated using the Illumina Stranded Total RNA Prep, and pooled libraries were sequenced on an Illumina NextSeq 550. As outlined in Arriaga et al., 2025[2], after sequencing, we removed 37 samples from further analysis due to missing information or insufficient RNA quality. Our resulting whole blood cohort consisted of 385 samples from 385 individuals.

Of all fibroblast samples, 134 of the total 139 samples originated from the Broad Center for Mendelian Genetics. cDNA libraries were generated using the Illumina TrueSeq Stranded mRNA Sample Prep Kit protocol and dual-indexed. The libraries were quantified after enrichment using Quant-iT PicoGreen (1:200 dilution). After normalizing samples to 5 ng/uL, the set was pooled and quantified using the KAPA Library Quantification Kit for Illumina Sequencing Platforms. Samples were then pooled and sequenced on Illumina Novaseq. For 108 samples, each run generated 101-bp paired-end reads, while for 30 samples, each run generated 151-bp paired-end reads. An eight-bp index barcode was generated for all samples.

The remaining five fibroblast samples, of which B1 was one, were prepared and sequenced by the UCLA Technology Center for Genomics and Bioinformatics using methods outlined in Lee et al., 2020[6]. Prior to sequencing, primary dermal fibroblasts were established from B1's skin biopsies. Cells were maintained at 37°C in a humidified atmosphere containing 5% CO_2 and cultured in standard growth medium composed of Dulbecco's Modified Eagle Medium supplemented with 10% fetal bovine serum and 1X Penicillin-Streptomycin-Glutamine. Adherent fibroblasts were then harvested from T75 flasks by enzymatic dissociation using TrypLE Express. Cells were washed with phosphate-buffered saline, pelleted by centrifugation, and prepared for RNA extraction. For RNA-sequencing, total RNA was isolated using the Maxwell RSC Instrument in conjunction with the Maxwell RSC simplyRNA Cells Kit, following the manufacturer's protocol. RNA concentration was measured using the Qubit Fluorometer, and RNA integrity was assessed with the Agilent Bioanalyzer. RNA-sequencing libraries were then sequenced using the Illumina NovaSeq 6000 platform, generating a minimum of 100 million paired-end reads (150 bp) per sample.

Pipeline

Transcriptome quality control and alignment

The computational pipelines for quality control and alignment 287 of the whole blood samples are outlined in Ungar et al., 2024[3], while the remaining 128 samples were preprocessed as described in Arriaga et al., 2025[2]. In short, we generated FASTQ files by demultiplexing BCL data using bcl2fastq (https://emea.support.illumina.com/sequencing/sequencing_software/bcl2fastq-conversion-software.html). The 287 samples analyzed in Ungar et al., 2024[3] were aligned to the hg38 human reference genome using STAR (2.8.4a)[7] and the GENCODEv35[8] primary genome annotations, while the remaining 128 samples were aligned using STAR (version=2.7.10a)[7] and GENCODEv39[8] primary genome annotations. Adapters for all 385 samples were removed, and reads were trimmed using cutadapt (version=2.4)[9] (https://github.com/marcelm/cutadapt) and optical duplicates removed using Picard (http://broadinstitute.github.io/picard).

FASTQ files for 134 of the fibroblast samples were generated from BCL data using IlluminaBasecallsToFastq (https://gatk.broadinstitute.org/hc/en-us/articles/9570268630683-IlluminaBasecallsToFastq-Picard) or BCL Convert (https://support.illumina.com/sequencing/sequencing_software/bcl-convert.html). The DRAGEN RNA pipeline from the Illumina DRAGEN Bio-IT Platform (https://support-docs.illumina.com/SW/dragen_v42/Content/SW/DRAGEN/TPipelineIntro_fDG.htm) was used with *--enable-duplicate-marking* set to true, allowing for duplicate read notation. PolyG and polyA tails were trimmed using *--soft-read-trimmers polyg, polya*. The minimum number of polyA bases required for trimming was set using *--trim-polya-min-trim* 20, and polyg soft trimming is enabled by default. Ribosomal RNA was removed using *--rrna-filter-enable=true*. The FASTQ files were then aligned to the hg38 human reference genome using STAR (version=2.7.10b)[7] in the two-pass mode and the GENCODEv39[8] primary genome annotation. For all 134 fibroblast samples, adapters were removed, and reads were trimmed using the Illumina DRAGEN Bio-IT Platform and optical duplicates removed using Picard (http://broadinstitute.github.io/picard).

RNA processing for the remaining 5 fibroblast samples is outlined in Lee et al., 2020[6]. Raw FASTQ files of the five fibroblast samples processed at UCLA were aligned to the GRCh37 human reference genome using STAR (version 2.5.2b)[7] with default parameters and GENCODEv19[8] primary genome annotation. Two-pass mapping was performed to maximize alignments across novel junctions. Depletion of ribosomal RNA was evaluated using BWA-mem[10] by calculating the portion of read pairs that aligned to references for the complete sequences of 5S, 12, 16S, 18S, and 28S ribosomal RNAs. Picard (http://broadinstitute.github.io/picard) performed duplicate marking, and additional quality metrics were generated via RNA-SeQC v1.1.8[11]. Single-nucleotide variant calls from genome sequencing were compared to the RNA-sequencing data to ensure correct sample identity.

Splicing outlier calling

Following guidelines from FRASER[12], we generated splicing outliers separately for the whole blood and fibroblast cohorts. The whole blood cohort was processed using FRASER (version=1.14.0)[12], while the fibroblast samples were processed using FRASER

(version=1.6.1)[12]. For both analyses, the default filterExpressionAndVariability settings were used; specifically, the minimum read count in at least one sample was 20, and the minimum $\Delta\Psi$ was 0.0.. The pipeline and conda environments for this analysis can be found at https://github.com/maurermaggie/Transcriptome_Wide_Splicing_Analysis/tree/main/FRASER_snakemake.

Analysis of minor intron retention

Arriaga et al., 2025[2] employed a transcriptome-wide approach examining intron retention outliers in minor intron-containing genes (MIGs) to identify individuals with rare, biallelic variants in the minor spliceosome. Their method identified five individuals with an excess (Z-score > 2) of significant intron retention outliers in MIGs. Four individuals harbored rare, biallelic variants in *RNU4ATAC*, a minor spliceosome snRNA previously associated with disease[13-15]. One individual, known in this study as A1, was found to have rare biallelic variants in *RNU6ATAC*. As minor spliceopathies are characterized by specific retention of U12-type introns, we refined the method in Arriaga et al., 2025[2] to filter for minor intron retention (as opposed to intron retention *of any type* in MIGs).

Following Arriaga et al., 2025[2], junctions were defined as significant if their $|\Delta\Psi|$ was greater than or equal to the default value suggested by FRASER[12] (0.3), and their adjusted p-value (q) after false discovery rate (FDR) correction was less than 0.05. $\Delta\Psi$ is a normalization metric from FRASER[12] that is similar to a z-score. For a given intron from a specified sample, $\Delta\Psi$ measures the difference between the observed and expected Ψ value.
The resulting significant ($q < 0.05$ and $|\Delta\psi| >= 0.3$) outliers were then filtered to only those of type θ, which captures partial or full intron retention. In order to filter for minor intron retention, we used the Homo_sapiens_intron download from the Minor Intron Database[16]. (https://midb.pnb.uconn.edu/return_downloads.php?species_var=Homo+sapiens). This database contains information on introns of the following types: major-like, minor-like, minor, non-canonical, major-hybrid, and minor-hybrid. We filtered to only introns with an intron_class of minor, then selected the following fields: gene_symbol, ensembl_gene_id, intron_start, and intron_end. We then filtered the significant θ introns for those whose gene name, Ensembl ID[17]

matched the first two aforementioned fields, and whose intron start and end matched the latter two fields ± 1. Similar to Arriaga et al., 2025[2], we defined individuals as having an excess of minor intron retention outliers if they contained a number of minor intron retention outliers greater than two standard deviations above the mean. The code used to filter our FRASER[12] outliers can be found at: https://github.com/maurermaggie/Transcriptome_Wide_Splicing_Analysis/tree/main/run_results_phenotype_paper.

References

1. Splinter K, Adams DR, Bacino CA, Bellen HJ, Bernstein JA, Cheatle-Jarvela AM, et al. Effect of Genetic Diagnosis on Patients with Previously Undiagnosed Disease. N Engl J Med. 2018 Nov 29;379(22):2131–9. Available from: https://doi:10.1056/NEJMoa1714458

2. Arriaga MT, Mendez R, Ungar RA, Bonner DE, Matalon DR, Lemire G, et al. Transcriptome-wide outlier approach identifies individuals with minor spliceopathies. Am J Hum Genet [Internet].2025 Oct 2;112(10):2458-2475. Available from: https://doi:10.1016/j.ajhg.2025.08.018

3. Ungar RA, Goddard PC, Jensen TD, Degalez F, Smith KS, Jin CA, et al. Impact of genome build on RNA-seq interpretation and diagnostics. Am J Hum Genet. 2024 July 11;111(7):1282–300. Available from: https://doi:10.1016/j.ajhg.2024.05.005

4. Cummings BB, Marshall JL, Tukiainen T, Lek M, Donkervoort S, Foley AR, et al. Improving genetic diagnosis in Mendelian disease with transcriptome sequencing. Sci Transl Med. 2017 Apr 19;9(386):eaal5209. Available from: https://doi:10.1126/scitranslmed.aal5209

5. Amar D, Nicole RG, Pierre MJ-B, Dam B, Surendra D, Courtney D, et al. Temporal dynamics of the multi-omic response to endurance exercise training. Nature. 2024 May;629(8010):174–183. Available from: https://doi:10.1038/s41586-023-06877-w

6. Lee H, Huang AY, Wang LK, Yoon AJ, Renteria G, Eskin A, et al. Diagnostic utility of transcriptome sequencing for rare Mendelian diseases. Genet Med Off J Am Coll Med Genet. 2020 Mar;22(3):490–9.

7. Dobin A, Davis CA, Schlesinger F, Drenkow J, Zaleski C, Jha S, et al. STAR: ultrafast universal RNA-seq aligner. Bioinforma Oxf Engl. 2013 Jan 1;29(1):15–21. Available from: https://doi:10.1093/bioinformatics/bts635

8. Frankish A, Diekhans M, Jungreis I, Lagarde J, Loveland JE, Mudge JM, et al. GENCODE 2021. Nucleic Acids Res. 2021 Jan 8;49(D1):D916–23. Available from: https://doi:10.1093/nar/gkaa1087

9. Martin M. Cutadapt removes adapter sequences from high-throughput sequencing reads. EMBnet.journal [Internet]. 2011 Oct 2;17, 10–12. Available from: https://doi:10.14806/ej.17.1.200

10. Li H, Durbin R. Fast and accurate short read alignment with Burrows-Wheeler transform. Bioinforma Oxf Engl. 2009 July 15;25(14):1754–60. Available from: https://doi.org/10.1093/bioinformatics/btp324

11. DeLuca DS, Levin JZ, Sivachenko A, Fennell T, Nazaire MD, Williams C, et al. RNA-SeQC: RNA-seq metrics for quality control and process optimization. Bioinforma Oxf Engl. 2012 June 1;28(11):1530–2. Available from: https://doi.org/10.1093/bioinformatics/bts196

12. Mertes C, Scheller IF, Yépez VA, Çelik MH, Liang Y, Kremer LS, et al. Detection of aberrant splicing events in RNA-seq data using FRASER. Nat Commun. 2021 Jan 22;12(1):529. Available from: https://doi.org/10.1038/s41467-020-20573-7

13. Merico D, Roifman M, Braunschweig U, Yuen RK, Alexandrova R, Bates A, et al. Compound heterozygous mutations in the noncoding RNU4ATAC cause Roifman Syndrome by disrupting minor intron splicing. Nat Commun. [Internet]. 2015 Nov 2;6, 8718. Available from: https://doi.org/10.1038/ncomms9718

14. Farach LS, Little ME, Duker AL, Logan CV, Jackson A, Hecht JT, et al. The expanding phenotype of RNU4ATAC pathogenic variants to Lowry Wood syndrome. Am J Med Genet [Internet]. 2018 Feb;.76, 465–469. Available from: https://doi.org/10.1002/ajmg.a.38581

15. Padgett RA, Shukla GC. A revised model for U4atac/U6atac snRNA base pairing. RNA [Internet]. 2002 Feb;125–128. Available from: https://doi.org/10.1017/s1355838202017156

16. Olthof AM, Hyatt KC, Kanadia RN. Minor intron splicing revisited: identification of new minor intron-containing genes and tissue-dependent retention and alternative splicing of minor introns. BMC Genomics [Internet]. 2019 Aug 30;20(1):686. Available from: https://doi.org/10.1186/s12864-019-6046-x

17. Dyer SC, Austine-Orimoloye O, Azov AG, Barba M, Barnes I, Barrera-Enriquez VP, et al. Ensembl 2025. Nucleic Acids Res [Internet]. 2025 Dec 4;53(D1), D948–D957. Available from: https://doi.org/10.1093/nar/gkae1071
